# Arresting Microphase Separation Encodes Material Mechanics by Sculpting Microarchitectures and Local Polymer Enrichment

**DOI:** 10.1002/adma.202512578

**Published:** 2025-12-10

**Authors:** Castro Johnbosco, Floris Dalenoord, Jarno Hiemstra, Yu Na, Alexis Wolfel, Cécile Bosmans, Christine Gering, Niels Willemen, Su Ryon Shin, Jeroen Leijten

**Affiliations:** ^1^ Leijten Laboratory Department of BioEngineering Technologies TechMed Centre Faculty of Science and Technology University of Twente Enschede 7500AE The Netherlands; ^2^ Division of Engineering in Medicine Department of Medicine Brigham and Women's Hospital Harvard Medical School Boston MA 02139 USA

**Keywords:** aqueous two‐phase system, mechanics, microarchitectures, multiscale materials, soft matter

## Abstract

Mechanical properties are central to material functionality. Although aqueous two‐phase systems (ATPS) can generate microarchitectures in soft materials such as hydrogels, their influence on mechanics, particularly toughness and energy dissipation, remains poorly understood. Here, diverse microarchitectures are systematically engineered within materials via ATPS‐induced local polymer enrichment, which yielding inverse globular, globular, and spinodal patterns, and revealing that each microarchitecture exhibits distinct mechanical behaviors. Most notably, spinodal hydrogel designs improve load distribution, increase fracture resistance, and promote efficient energy dissipation. These insights are used to develop and introduce single polymer phase separation (SPPS) as an innovative strategy to sculpt microarchitectures by tuning the ionic concentration, which overcomes traditional limitations of dual polymer systems. This novel approach enables scalable, low‐complexity, and chemically clean control over stiffness, toughness, and energy dissipation, independent of secondary polymers. Beyond mechanical advantages, spinodal architectures also support enhanced cell migration and biological activity. These findings demonstrate that microarchitectural design, rather than total polymer composition alone, dictates hydrogel mechanics. ATPS and SPPS provide robust and scalable methods to encode distinct mechanical and functional properties via microarchitecture variations into hydrogels, opening opportunities across tissue engineering, biofabrication, soft electronics, and food engineering.

## Graphical abstract

Microarchitecture sculpting via phase separation programs hydrogel mechanics by creating globular, inverse globular, and spinodal structures. Spinodal architectures deliver superior toughness and energy dissipation, while ionic modulation through single polymer phase separation offers a simple, scalable route to tune mechanical properties, complementing ATPS for diverse applications.

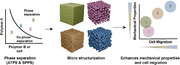

## 1 Introduction

Microarchitectures can encode functional properties in materials such as hydrogels, which have proven pivotal for various applications.^[^ ^1^, ^2^, ^3^ ^]^ Aqueous two‐phase systems (ATPS) have emerged as an innovative material structurization strategy to introduce highly sculpted yet naturally formed microarchitectures within engineered materials.^[^ ^4^, ^5^ ^]^ ATPS achieves this by facilitating phase separation of distinct polymers in fully aqueous environments, which is driven by molecular arrangement, surface tension, and viscosity differences.^[^ ^6^, ^7^ ^]^ This separation can result in island‐like (e.g., globular) or bicontinuous (e.g., spinodal) polymer networks microarchitectures, which can be stabilized via sol‐gel transitioning to yield ATPS hydrogels.^[^ ^8^ ^]^ These micro‐architected hydrogels exhibit unique isotropic patterns and structural organization that act in a function‐endowing manner for diverse applications.^[^ ^9^, ^10^ ^]^ For example, in tissue engineering, this microstructurization facilitates cell infiltration,^[^ ^11^ ^]^ unlike non‐structured nanoporous hydrogels, making them ideal scaffolds for therapeutic cell delivery, cell migration studies,^[^ ^12^ ^]^ and engineering of voluminous living tissues.^[^ ^13^ ^]^ Moreover, these microarchitectures can also provide enhanced sensitivity of functional surfaces for biosensing applications,^[^ ^14^ ^]^ and allow for improved material flexibility and mechanical adaptability for soft robotics and flexible electronics applications.^[^ ^15^ ^]^

Despite increasing interest in ATPS‐derived microarchitected hydrogels for diverse applications, the impact of ATPS‐driven microstructural organization on mechanical performance remained underexplored. Traditionally, studies have primarily focused on characterizing and controlling pore size as a determinant of construct functionality.^[^ ^10^, ^11^, ^16^, ^17^, ^18^ ^]^ A limited number of studies have reported enhanced bulk mechanical properties in ATPS systems^[^ ^4^, ^9^, ^19^ ^]^ but a comprehensive understanding of the physical characteristics, such as microarchitecture shape and connectivity on a construct's stiffness, toughness, and energy dissipation has remained wanted. Moreover, microarchitectures in ATPS hydrogels have predominantly been generated through dual polymer systems, where parameters such as molecular weight,^[^ ^20^ ^]^ entropy of mixing,^[^ ^21^ ^]^ crosslinking kinetics,^[^ ^18^ ^]^ and polymerization dynamics^[^ ^22^ ^]^ strongly influence structural outcomes. This dependence on dual polymers introduces complexity and leads to variability, limited reproducibility, and poor scalability. We anticipated that addressing this knowledge gap would yield a novel understanding of how these microstructural parameters govern the mechanics of ATPS materials, and would offer more predictable and reproducible approaches to produce function‐endowing microarchitectures to unlock more precise and tailored applications, which was anticipated to drive their use in next‐generation engineered materials.

Herein, we hypothesized that ATPS sculpting of microarchitectures would dictate the mechanical performance of bulk ATPS hydrogel systems in an architecture‐specific manner. To test this hypothesis, we employed poly(ethylene glycol) dimethacrylate (PEGDMA) and alginate, commonly used biopolymers specifically used for engineering IPN (interpenetrating polymer network) hydrogels. We demonstrated that at commonly reported concentrations, PEGDMA and alginate polymers consistently form ATPS hydrogels via local enrichment of each polymer. We then systematically varied the polymer content within these hydrogels to map the formation of specific microarchitectures (i.e., inverse globular, globular, and spinodal configurations). All architectures could be isotopically patterned by adjusting the ratios of the immiscible polymer solutions, allowing for precise control over structural features such as strut width, thickness, and length. Interestingly, spinodal architectures endowed hydrogels with enhanced mechanical properties, including increased Young's modulus and toughness, compared to globular and inverse globular configurations. Under unconfined compression, spinodal microarchitectures enabled remarkable geometrical compression and shape recovery by endowing elastic hydrogels with a stress‐dissipative nature, which was fully encoded in its unique microarchitecture. Moreover, encoded microarchitectures were demonstrated to determine cell migration with spinodal microarchitectures providing an optimal environment for cell migration enhancement, which further confirms ATPS' potential applications for the engineering of living matter. To remove dual polymer complexity, we introduced an innovative yet simple and scalable strategy to encode microarchitectures in hydrogels using single polymer phase separation (SPPS). This was achieved using deterministic addition of NaCl to predictably sculpt various microarchitectures that significantly enhanced the overall mechanical properties of sculpted hydrogels. These findings highlight the underexplored impact of microarchitecture on the mechanics of bulk ATPS and SPPS hydrogel systems, which have the potential to expand the utility of material microstructurization via spatially organizing local material enrichment that can be beneficial across diverse fields of material science. Notably, the introduction of salt as a replacement for a second polymer for phase separation positions SPPS as a robust, low‐cost, and scalable approach to naturally sculpt microarchitectures into common materials, including hydrogels that can be leveraged for various applications, including bioengineering, flexible electronics, soft robotics, and architected soft matter.

## 2 Results and Discussion

### 2.1 Hydrogel Microarchitecture Sculpted by ATPS Governs Mechanical Properties

**Figure 1:**
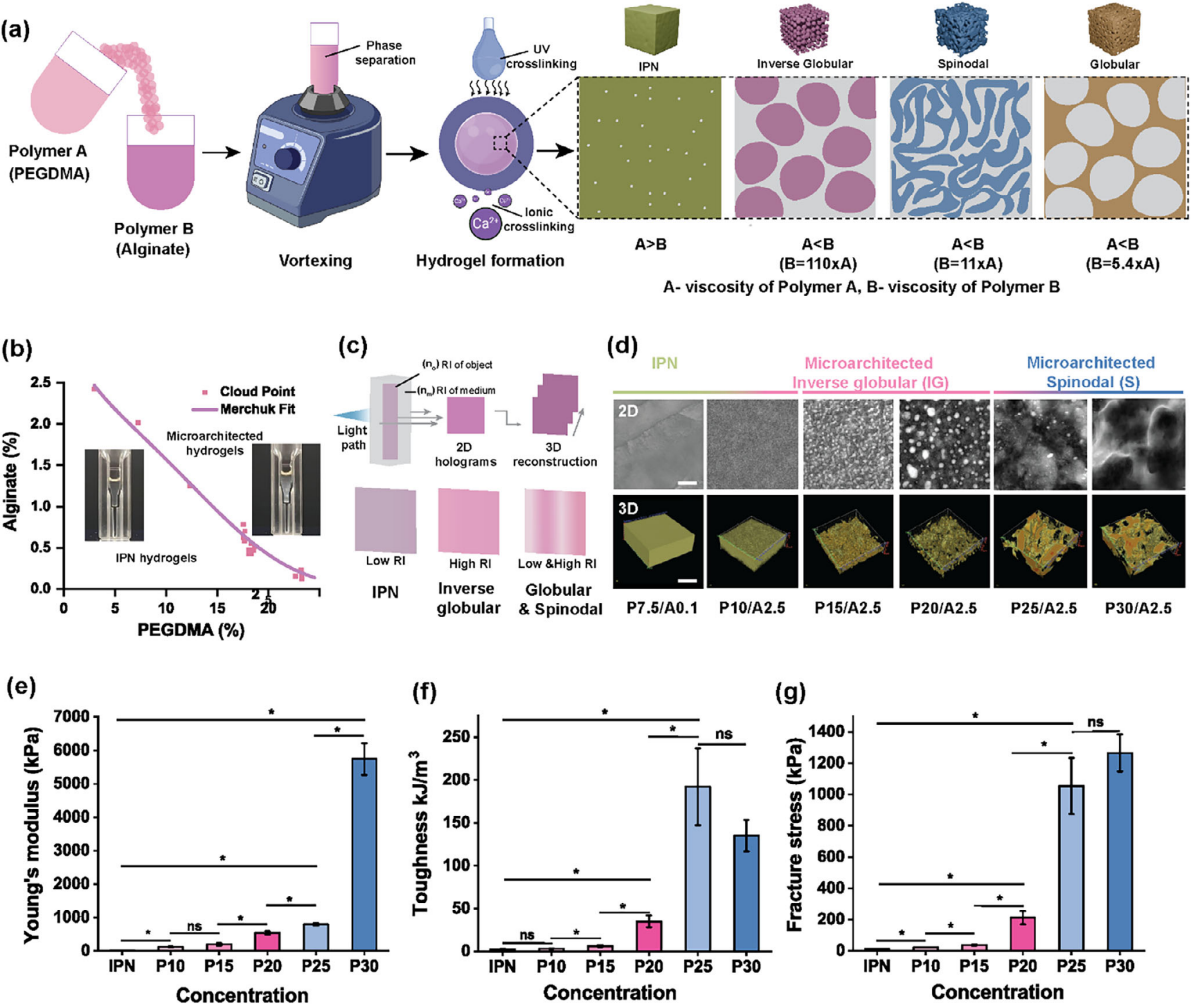
Characterization of polymer blends and its microarchitectures corresponding to variable mechanical properties, a) Schematic depiction of polymer immiscibility governing phase separation between (PEGDMA and alginate) leading to various microarchitectures (IPN, inverse globular, spinodal, and globular) modulated by viscosity difference. b) Binodal curve of PEGDMA and alginate, all concentrations are depicted in weight‐to‐weight percentages, photographs of a vial consisting of phase‐separated and non‐phase‐separated formulations. c) Principle of holotomography to detect differences in phase‐separated polymer phases using refractive index differences. d) Holotomographic images (2D and 3D) of differently microstructured hydrogels, including (non‐microarchitectured IPN, microarchitectured inverse globular, and microarchitectured spinodal) hydrogels (Scale bar is 100 µm). For example, (P represents PEGDMA and A represents alginate, followed by their respective concentration). e) Young's modulus f), toughness, and g) fracture stress of various microarchitectures obtained by varying the concentration of PEGDMA with constant alginate concentration 2.5% (n = 3 samples per condition).^*^ indicates statistical significance at *p*<0.05, as determined by Kruskal–Wallis ANOVA. All data are presented as mean ± s.e.m.

Poly(ethylene glycol) (PEG)‐based polymers are widely recognized for their phase separation properties, making them an excellent choice for creating polymer blends with distinct molecular characteristics.^[^ ^23^, ^24^ ^]^ PEG is known to phase separate with numerous polymers, including other commonly used polymers such as dextran, alginate, gelatin, and hyaluronic acid.^[^ ^20^, ^25^ ^]^ In this study, we selected poly(ethylene glycol) dimethacrylate (PEGDMA) as a representative synthetic biocompatible polymer and combined it with alginate, a natural biocompatible polymer. We specifically chose PEGDMA‐alginate hydrogels as this material combination is known to possess augmented mechanical performance owing to its supposed interpenetrating network (IPN) design.^[^ ^26^ ^]^ However, it has also been reported that polyethylene‐based polymer solutions phase separate from alginate when mixed at sufficiently high concentrations.^[^ ^25^ ^]^ We therefore set out to evaluate whether these emerging properties truly arise from an IPN mechanism or whether they stem from currently unknown effects caused by phase separation. We evaluated how viscosity as a descriptor can be tuned between these polymers to achieve various microarchitectural configurations (**Figure** **1**a).

To identify phase separation, critical concentrations at which PEGDMA and alginate homogeneous solutions transform into phase‐separated solutions were systematically mapped using cloud point titration^[^ ^27^ ^]^ (Figure 1b). Subsequently, we explored the use of holotomography to characterize the microstructurization of distinct PEGDMA‐alginate hydrogel compositions. This non‐invasive imaging technique quantitatively determines refractive index differences in a voxelated and label‐free manner (Figure 1c), avoiding the use of fluorescent dyes that could disrupt the network architecture. Although the technique is traditionally used to visualize cell membranes, nuclei, and mitochondria in a label‐free manner,^[^ ^28^ ^]^ we revealed that this imaging technique is also highly suitable for 3D resolved visualization of spatially distinct topological properties and microstructural features of polymeric multimaterials, including architected phase separations.

Using this approach, we observed substantial microstructural variations in hydrogels composed of PEGDMA and alginate when the PEGDMA concentration was increased from 10% to 30%, while maintaining a constant alginate concentration (Figure 1d). In contrast, hydrogels composed solely of PEGDMA or alginate did not exhibit these patterns (Figure S1, Supporting Information), indicating that the microarchitecture formation is specific to phase‐separated systems. Quantitative refractive intensity analysis showed that spinodal hydrogels (P25, P30) exhibited distinct polymer‐rich and polymer‐sparse phases, reflected by a leftward intensity shift compared to IPN hydrogels. Inverse globular hydrogels (P10–P20) displayed more homogeneous polymer distribution with fewer sparse regions, resulting in higher overall intensity. The polymer‐rich/sparse ratio further distinguished architectures, being ≥1 for spinodal, ≤0.5 for inverse globular, and <0.1 for IPN hydrogels (Figure S2, Supporting Information). Holotomography confirmed these findings, demonstrating concentration‐dependent microarchitecture formation via phase separation of the polymer networks.

We then hypothesized that, in addition to the local polymer concentration, differences in microarchitecture would influence the mechanical properties of hydrogels. Rheological analysis demonstrated that spinodal hydrogels exhibited significantly enhanced mechanical properties, with a transition in microarchitecture from inverse globular to spinodal associated with a significant increase in the hydrogel's Young's modulus (Figure 1e). Specifically, while an inverse globular architecture (e.g., P20) and a spinodal architecture with still some inverse globular features (e.g., P25) exhibited a Young's modulus of 541.50 ± 58.29 kPa and 796 ± 37 kPa respectively, a full spinodal architecture (e.g., P30) showed an order of magnitude increase in stiffness (e.g., 5741 ± 476 kPa). Moreover, transitioning from inverse globular to spinodal architecture is associated with a five‐fold increase in toughness, with spinodal hydrogels reaching values of up to 192 ± 45 kJ m^−^^3^ (Figure 1f). The tougher nature of spinodal architectures was further corroborated by fracture stress measurements, which increased in spinodal hydrogels (e.g., P25; 1055 ± 179 kPa) than inverse globular hydrogels (e.g., P20; 213 ± 43 kPa) (Figure 1g). These findings consistently highlight a clear relationship between microarchitectures and mechanical properties in PEGDMA‐alginate hydrogels. Notably, spinodal hydrogels demonstrated superior mechanical robustness, strength, and toughness compared to their inverse globular counterparts, underscoring the importance of microarchitecture to yield mechanically advanced material systems.

### 2.2 Microarchitecture Sculpting via Local Polymer Enrichment Guided Mechanical Properties Beyond Material Dependence

**Figure 2:**
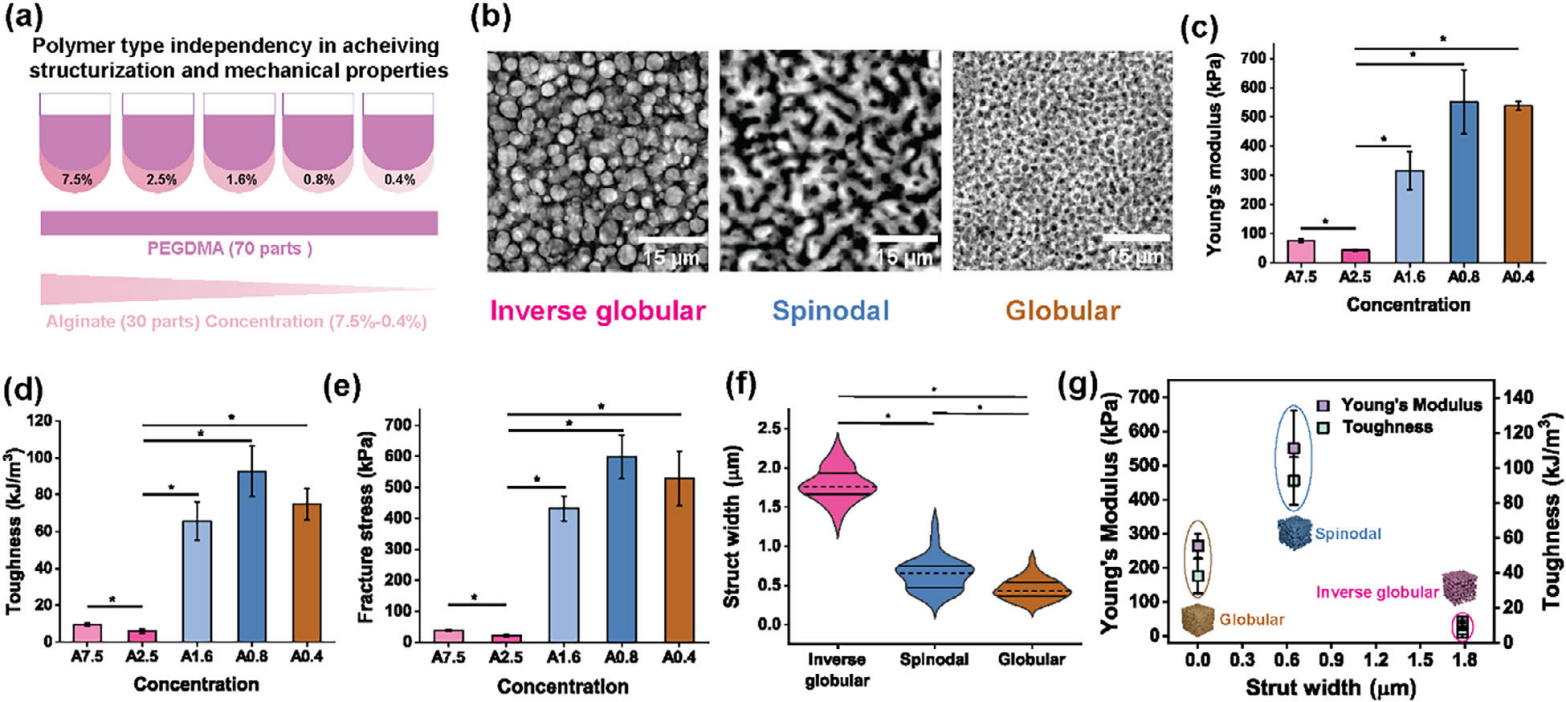
a) Characterization of polymer type independence on the microstructurization and their mechanical properties by varying alginate concentrations. b) Representative Holotomographic images of different microstructured hydrogels (inverse globular, spinodal & globular). c) Young's modulus, d) Toughness, and e) Fracture Stress of variable concentrations of alginate from 7.5% to 0.4% with fixed PEGDMA concentration 25% to determine polymer type dependency in microarchitecture formation (n = 3 samples per condition). ^*^ indicates statistical significance at *p*<0.05, as determined by Kruskal–Wallis ANOVA. f) Strut size calculated from Holotomographic images using ImageJ to determine the strut size variations within different microarchitecture patterns inside (inverse globular, spinodal & globular) hydrogels (n≥98 points from 3 different images). ^*^ indicates statistical significance at *p*<0.05, as determined by one‐way ANOVA. g) Young's Modulus and toughness comparisons with the strut size of various microarchitecture patterns that correlate the mechanical properties with microarchitecture dimensions (n = 3 samples per condition). All data are presented as mean ± s.e.m.

To confirm that mechanical properties arose from microarchitecture rather than total polymer concentration, we varied PEGDMA/alginate mixing ratios while keeping concentrations constant. Spinodal hydrogels (e.g., 70/30) consistently outperformed inverse globular hydrogels (e.g., 60/40), exhibiting markedly higher shear modulus, yield stress, Young's modulus, toughness, and fracture stress. Holotomography confirmed that higher PEGDMA ratios formed spinodal structures, whereas lower ratios produced inverse globular architectures. These results demonstrate that hydrogel mechanics are governed by microarchitecture type, independent of material concentration (Figure S3, Supporting Information). To evaluate the microarchitecture formation is not purely a material‐driven factor that might obscure the understanding of microarchitecture formation and its effects within hydrogels, we also explored varying alginate concentrations while keeping PEGDMA concentration and all volume ratios constant (**Figure** **2**a). This approach minimized multilevel complexities and allowed for deeper exploration of the 70/30 ratio, of which 25% PEGDMA and 0.8% alginate yielded spinodal‐enhanced mechanical properties. Strikingly, altering alginate concentrations alone resulted in the sculpting of three distinct microarchitectures: inverse globular, spinodal, and globular structures (Figure 2b). Mechanical analysis of the distinctly architected hydrogels demonstrated a clear correlation between microarchitecture and mechanical properties. Spinodal hydrogels exhibited significantly higher Young's modulus (551 ± 109 kPa) followed by globular hydrogels (538 ± 15 kPa), and both significantly outperform inverse globular hydrogels (43 ± 3.8 kPa) (Figure 2c). Toughness measurements further underscored the mechanical superiority of spinodal hydrogels (93 ± 14 kJ m^−^^3^), being a remarkable 13‐fold higher than inverse globular hydrogels (6 ± 1.2 kJ m^−^^3^) (Figure 2d). Spinodal hydrogels were also characterized by a 27‐fold enhancement in fracture stress (598 ± 71 kPa) compared to inverse globular hydrogels (22 ± 3.8 kPa) (Figure 2e). Interestingly, both spinodal and globular hydrogels shared comparable mechanical properties, suggesting a more potent role for phase‐separated yet continuous and interconnected structural organization than obtaining a bicontinuous design. Indeed, the absence of this organization in inverse globular hydrogels is consistently associated with inferior mechanical properties. To understand how the overall mechanical properties are determined by microarchitecture variations, strut width measurements were then performed to correlate architectural differences with bulk hydrogel mechanics. Inverse globular hydrogels exhibited significantly larger strut size (1.78 ± 0.2 µm) than spinodal (0.64 ± 0.02 µm) and globular (0.45 ± 0.14 µm) hydrogels (Figure 2f). Notably, the smaller strut architecture of spinodal and globular hydrogels corresponded with higher Young's moduli (551 ± 109 kPa and 538 ± 15 kPa, respectively) and greater toughness (92.66 ± 13.65 kJ m^−^^3^ for spinodal hydrogels) (Figure 2g). However, to substantiate that these microarchitectural differences are the critical factor to enhance overall bulk mechanics, we performed optical interferometry‐based nano‐indentation analysis on different microarchitected hydrogels. Spinodal and globular hydrogels exhibited higher Youngs modulus than the inverse globular architecture, showing distinct polymer‐rich and polymer‐sparse regions (Figure S4, Supporting Information). Our results clearly demonstrate that it is not merely local micromechanical variations but rather the topology of the sculpted microarchitectures, specifically their size, shape, and connectivity, that dictates the bulk mechanical properties of phase‐separated hydrogels. To further elucidate whether the ATPS‐induced mechanical changes were caused by the encoded microarchitecture, the altered local polymer concentration, or both, we compared a spinodal PEGDMA hydrogel with a nanoporous PEGDMA hydrogel of which the polymer concentration was matched to that of the local polymer enrichment of the spinodal hydrogel (i.e., identical local polymer concentration, but distinct total polymer content) (Figure S5, Supporting Information). This revealed that spinodal microarchitectures enable energy dissipation, local polymer enrichment increases fracture stress, and the spinodal microarchitecture and polymer enrichment both increase material toughness. Consequently, local polymer enrichment and specific microarchitectural configuration synergistically enhance the mechanical properties of ATPS‐sculpted hydrogels.

To test the generality of this approach, we evaluated other polymers, including gelatin methacrylate (GelMA), hyaluronic acid, and dextran‐tyramine (DexTA). Holotomography confirmed that these systems also produced defined microarchitectures at specific concentrations. Replacing alginate with GelMA further showed that PEGDMA–GelMA spinodal hydrogels possessed superior mechanical properties (Figure S6, Supporting Information). Importantly, microarchitecture formation remained consistent across variations in temperature, crosslinking intensity, and mixing time, underscoring the robustness and reproducibility of this strategy for diverse applications (Figure S7, Supporting Information). The consistent and reproducible correlation between defined microarchitectural patterns (e.g., spinodal versus globular) and distinct enhancement in bulk mechanics reflected in the Young's modulus, toughness, and fracture stress values suggested that topology was the dominant factor. Collectively, these findings establish that microarchitectures sculpted via local polymer enrichment act as a primary and deterministic regulator of hydrogel mechanics.

### 2.3 Sculpting Microarchitectures Dictates Mechanical Performance, Including Shape Recovery, Stretchability, and Energy Dissipation in Engineered ATPS Hydrogels

**Figure 3:**
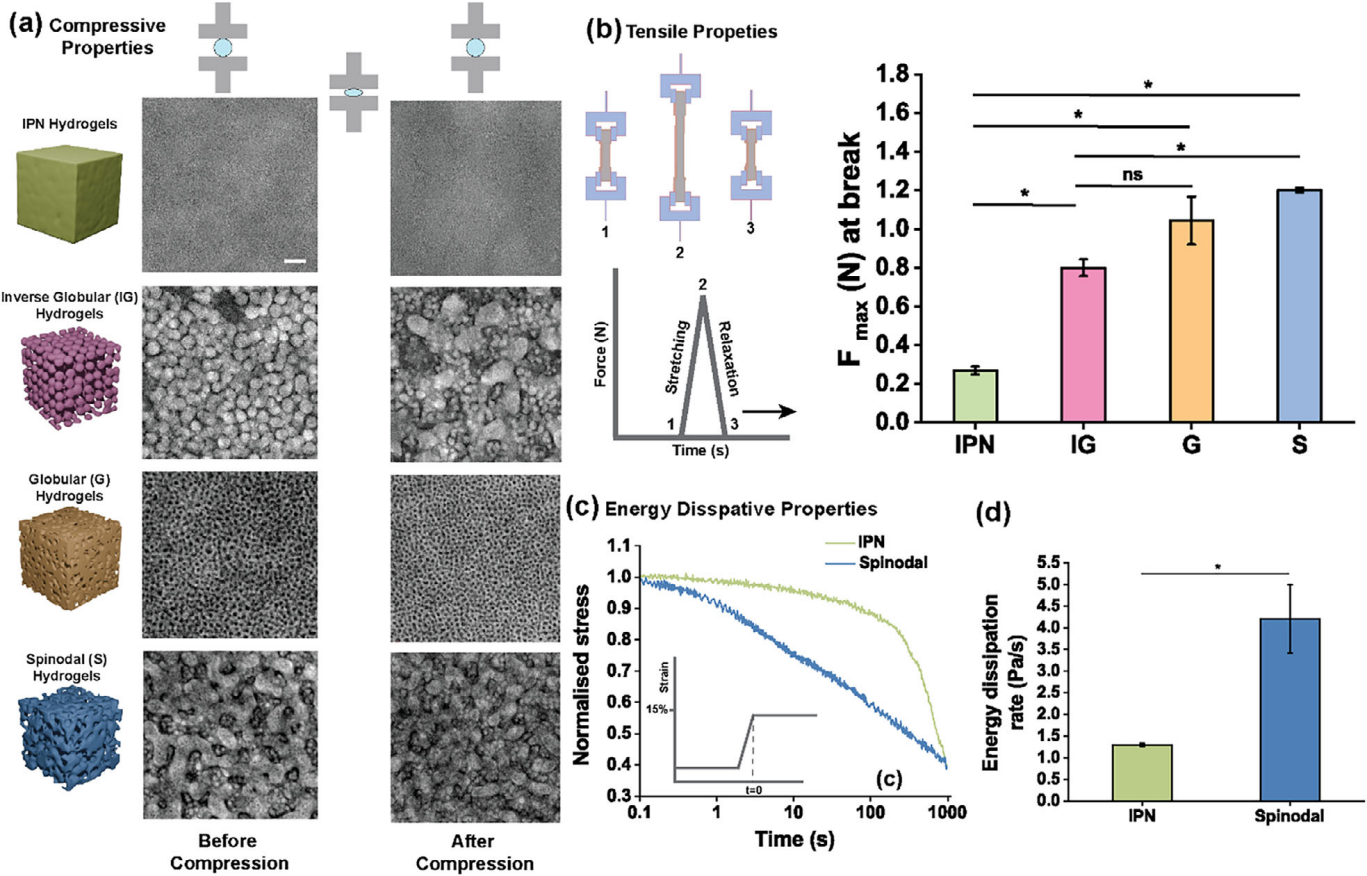
a) Representative Holotomographic images of various microstructure patterns of inverse globular, spinodal & globular hydrogels obtained before compression, and post 50% compression to determine the geometric recovery ability of these microstructures inside these hydrogels (n = 3 individual images from 2 independent samples). Scale bar indicates 5 µm. b) Cyclic stretching measurement to determine the effect of microarchitectures of hydrogels' mechanical integrity under cyclic deformation to determine F_max_ at the point of rupture from cyclic stretching. Energy dissipation properties of microarchitected hydrogels. c) Energy dissipation curves represented as normalized stress values for IPN and microarchitected spinodal hydrogels. d) Energy dissipation rates were calculated as a decay of stress over time after 1 s of constant strain for IPN and hydrogels. (n = 3 samples per condition). ^*^ indicates statistical significance at *p*<0.05, as determined by Kruskal–Wallis ANOVA. All data are presented as mean ± s.e.m.

To investigate whether these microarchitectures and local polymer‐enriched hydrogels also exerted a distinct ability to withstand mechanical loading, we performed in situ holotomographic imaging under strain‐controlled compression. While spinodal and globular hydrogels retained structural integrity up to 50% strain, inverse globular hydrogels exhibited irreversible shape deformation already at 30% strain (Figure S8, Supporting Information) and developed macroscopic cracks at higher strains (**Figure** **3**a). Furthermore, we evaluated how these microarchitectural contributions could influence the stretchability and flexibility of hydrogels. We performed cyclic stretching and energy dissipation tests showed that spinodal hydrogels had superior stretchability and tensile strength compared to IPN hydrogels. IPN hydrogels consistently exhibited the weakest performance, lower than spinodal, globular, and inverse globular architectures. F_max_ at break further confirmed this trend: spinodal hydrogels reached 1.20 ± 0.01 N, inverse globular 0.79 ± 0.04 N, while IPN hydrogels had the lowest value at 0.26 ± 0.02 N (Figure 3b). These results demonstrate that microarchitecture is a decisive factor in tuning hydrogel mechanics, enabling both strengthening and controlled weakening. This framework highlights the potential to tune hydrogel mechanics via microstructural design, offering new avenues for engineering hydrogels with designer mechanical properties.

As hydrogels with spinodal architectures were able to withstand cyclical loading and unloading via tensile measurements, we reasoned that the microarchitecture might endow hydrogels with energy‐dissipating properties owing to the periodic spatial distribution of polymer‐rich and polymer‐sparse domains. In biological tissues, energy dissipation through energy dissipation is a common phenomenon, driven by their inherent viscoelasticity.^[^ ^29^ ^]^ Tissues under mechanical stress relax in a time‐dependent manner, dissipating energy via their nanoscale or microscale architectures to maintain structural integrity.^[^ ^30^ ^]^ Yet designing materials capable of both high energy absorption and dissipation has remained challenging, as irreversible deformations hinder effective energy distribution.^[^ ^31^ ^]^ To this end, we investigated whether spinodal hydrogels exhibit energy‐dissipating properties by applying a constant strain while measuring stress in the hydrogel over time. This revealed that spinodal hydrogels exhibited far faster energy dissipation (e.g., τ_1_^/^_2_ of ≈283 s) than IPN hydrogels (e.g., τ_1_^/^_2_ of ≈729 s) (Figure 3c). We further evaluated the energy dissipation rate to determine the percentage of stress decay at 1s, which revealed that spinodal hydrogels exhibited ≈4‐fold higher energy dissipation rate (4.207 ± 0.78) % per second compared to IPN hydrogels (1.29 ± 0.03) % per second (Figure 3d). To investigate whether microarchitecture via ATPS‐driven local polymer enrichment alone could confer hydrogels with an energy dissipative nature, we generated PEGDMA–alginate spinodal hydrogels and subsequently enzymatically leached all alginate out of the hydrogels, leaving microporous PEGDMA hydrogels that were solely stabilized by static covalent crosslinks. Remarkably, these leached spinodal hydrogels showed much faster energy dissipation (τ_1_/_2_ ≈10.4 s) and a threefold higher dissipation rate (13.8 ± 0.9%/s) compared to non‐leached counterparts (Figure S9, Supporting Information). These results highlight that alginate is primarily required only to drive phase separation, creating well‐defined locally enriched PEGDMA spinodal architectures that determine hydrogel mechanics. Spinodal hydrogels, even with a single polymer, uniquely couple high stiffness, toughness, and load‐bearing capacity with rapid, tunable energy dissipation, establishing ATPS microarchitecture sculpting as a powerful strategy to engineer elastic hydrogels with exceptional mechanical performance.

### 2.4 Single Polymer Phase Separation (SPSS) Sculpts Microarchitectures that Alter the Mechanical Nature of Hydrogels

**Figure 4:**
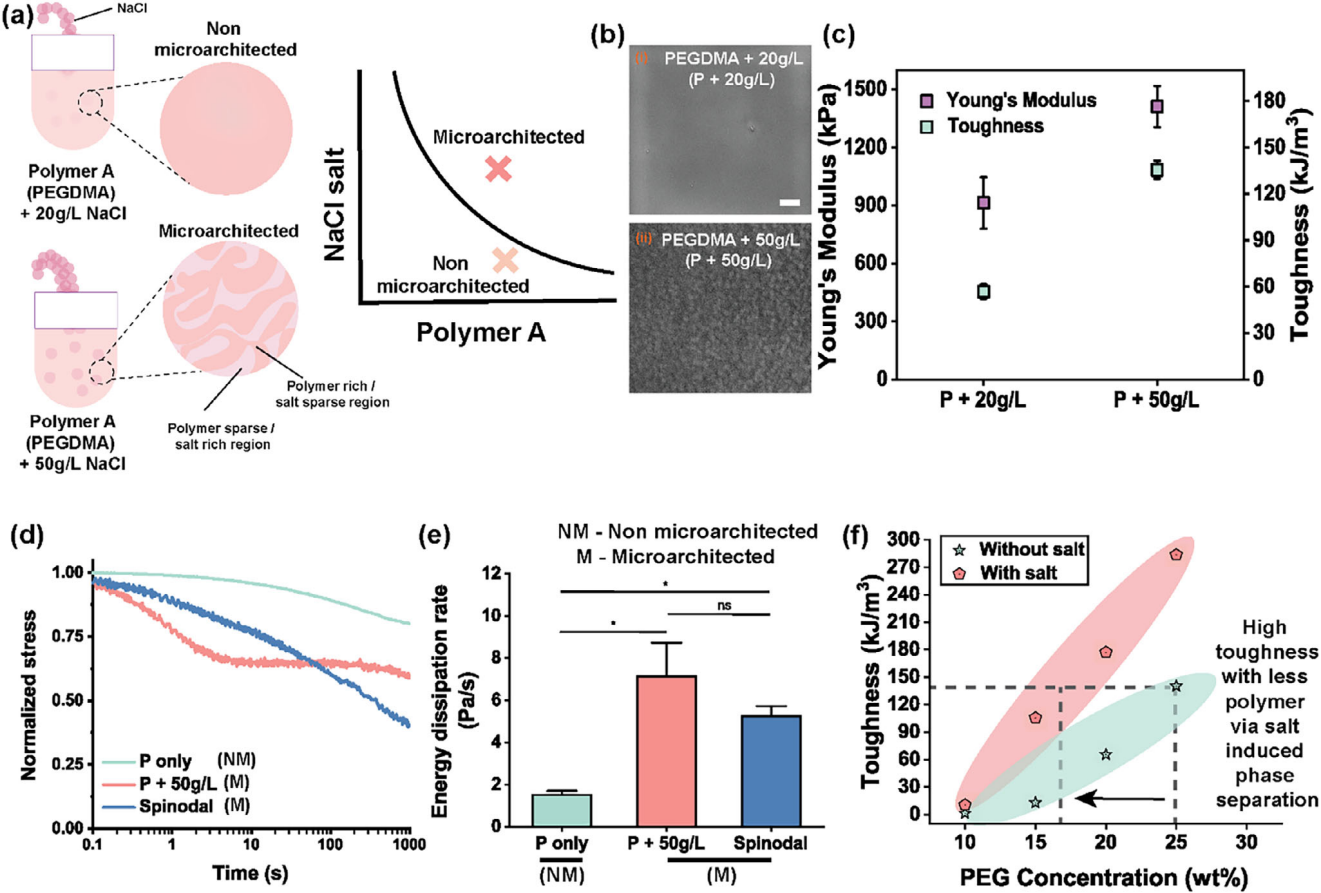
a) Single polymer phase separation using NaCl can sculpt microarchitectures into hydrogels. b) Holotomographic representative images of PEGDMA with (i) 20 g L^−1^ salt (non‐phase separated) hydrogels and (ii) PEGDMA phase separated by 50 g L^−1^ salt. Bulk mechanical properties. Scale bar indicates 5 µm. c) Young's modulus, and toughness of non‐structured hydrogels (P+20 g L^−1^)& micro‐architected hydrogels (P + 50 g L^−1^). d) Representative energy dissipation curves and e) energy dissipation rates of PEGDMA‐only hydrogels (non‐micro‐architected), salt‐induced microarchitecture spinodal hydrogels, and spinodal hydrogels formed by PEGDMA and alginate. f) Distribution clouds to determine the local polymer enrichment that enhanced the bulk mechanical property toughness values with PEGDMA only and PEGDMA + 50 g L^−1^ salt samples. (n = 3 samples per condition). ^*^ indicates statistical significance at *p*<0.05, as determined by Kruskal–Wallis ANOVA. All data are presented as mean ± s.e.m.

As all our previous experiments relied on multi‐material systems in which microarchitectures were sculpted by varying polymer concentration or ratios, the potential effect of these compositional variations (i.e., the individual contribution of each component) on mechanical performance could not yet be fully excluded. To enhance the scalability and minimalistic design aspect, reducing the complexity, we next designed an experiment leveraging single polymer phase separation (SPPS) to generate micro‐architected hydrogels. This was achieved by the introduction of ions during photopolymerization that drove polymeric chain dehydration by increased local concentration of Na^+^ and Cl^−^ ions.^[^ ^32^, ^33^ ^]^ Following Flory‐Huggins theory,^[^ ^34^ ^]^ addition of NaCl to a polymeric (i.e., PEGDMA) solution with water molecules effectively weakens the affinity of PEGDMA toward water. Hence, the polymer solvent interaction increases, forcing PEGDMA to decompose into polymer‐rich and polymer sparse phases. The interfacial tension between these two phases minimizes the Gibbs free energy,^[^ ^35^ ^]^ which determines the overall microarchitecture formation with defined void spaces. Inspired by this underexplored phase‐separation approach based on salts in liquid‐liquid systems,^[^ ^36^, ^37^ ^]^ we adjusted the sodium chloride concentration in 25% PEGDMA solutions to either 20 or 50 g L^−1^ (**Figure** **4**a). Adjusting the salt concentration shifted the binodal curve to either achieve or avoid liquid‐liquid phase separation like in our otherwise identical material systems (Figure 4b). Mechanical analysis revealed that even when using a single polymer, the Young's modulus of spinodal architected hydrogels was ≈1.5 times as high (1411.26 ± 106.10 kPa) compared to their nanoporous counterparts (912.52 ± 132.90 kPa. Similarly, formation of salt‐induced spinodal microarchitecture increased hydrogel toughness by 82.29% (271.02 ± 11.67 kJ m^−3^) approximately (Figure 4c). Of note, mechanical analysis of PEGDMA hydrogels with either 20 g L^−1^ or no salt revealed that introduction of low salt concentrations did not significantly affect the hydrogel's mechanical properties (Figure S10, Supporting Information).

These findings validate that phase separation‐induced sculpting of micro‐architectures via local polymer enrichment is essentially sufficient to enhance the bulk mechanical behavior of hydrogels, and in particular for spinodal microarchitectures. Moreover, salt‐induced SPPS endowed the otherwise fully elastic hydrogel with an energy‐dissipating nature as the primary relaxation time (τ_11_^/^_2_) was reduced upon introduction of a spinodal architecture (Figure 4d). Indeed, SPPS spinodal hydrogels were able to dissipate energy at a rate of 13.7 ± 5 Pa/s (Figure 4e). Remarkably, the toughness profiles of PEGDMA alone and PEGDMA with an optimal salt concentration demonstrate that the addition of salt to polymer precursor solutions can significantly enhance mechanical properties after crosslinking. Notably, a toughness of ≈140 kJ m^−^^3^, originally observed in 25% PEGDMA without salt, can be replicated using only 17% PEGDMA when salt is used to induce phase separation‐induced microstructurization (Figure 4f). Phase separation‐induced micro‐structurization, especially salt‐induced SPPS, compensates for reduced polymer content while enhancing toughness and energy dissipation, yielding mechanics comparable to dual‐polymer systems. These results highlight that phase separation enriches polymer‐rich domains while introducing complementary polymer‐sparse regions, thereby enhancing mechanical properties such as stiffness, toughness, and energy dissipation. This architecture enables superior mechanics with less total polymer compared to unstructured bulk systems, underscoring the critical role of microarchitecture rather than overall polymer concentration.

### 2.5 Hydrogel Microarchitectures Can Program Cell Migration Rates

**Figure 5:**
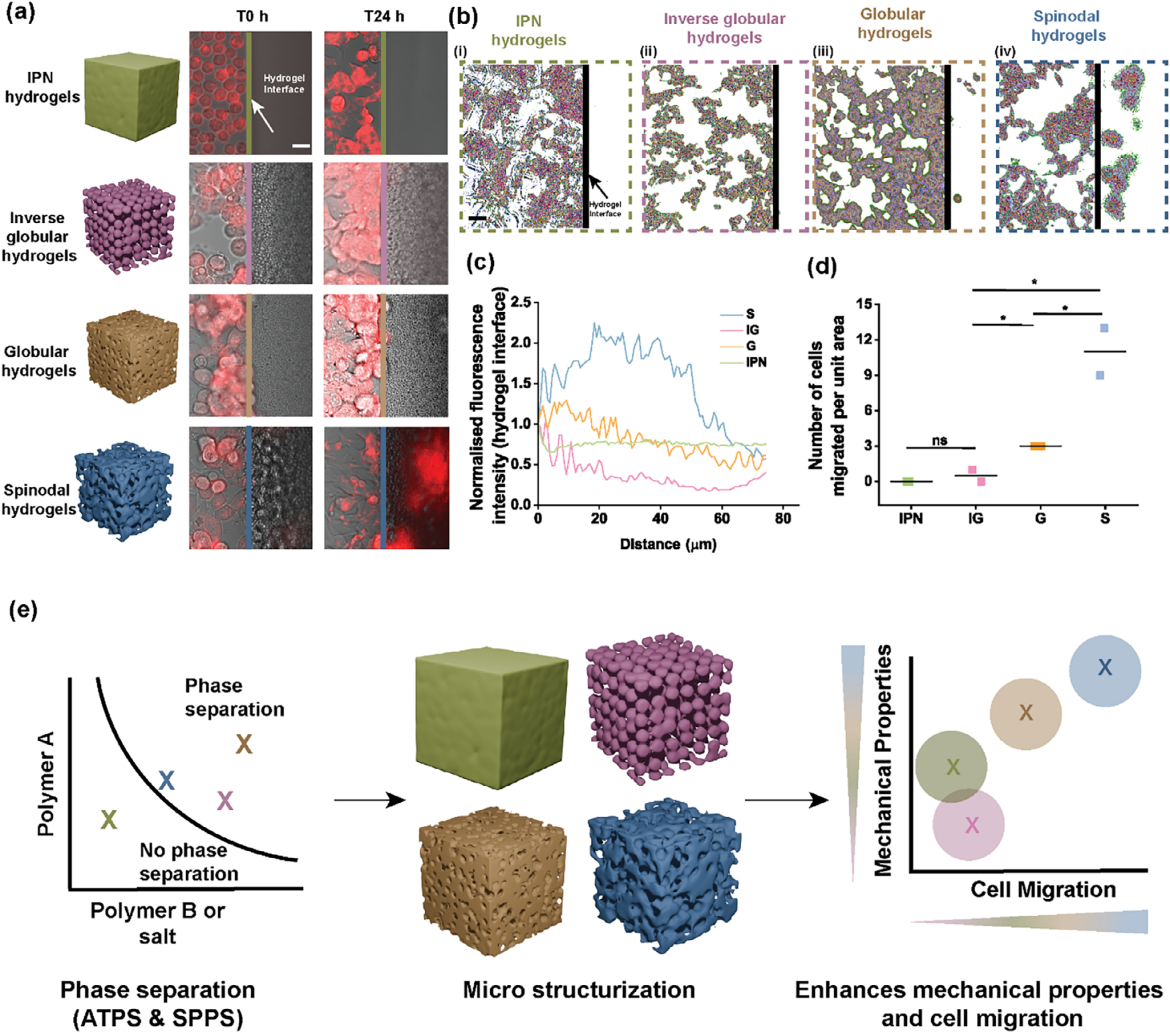
Microstructurization and its influence on cellular migration activity. a) Representative fluorescent confocal images of cell migration at the hydrogel interface at T0 and T24 time points post‐seeding of the cells. The fluorescent images were obtained to determine the migration capability of cells inside different microstructured hydrogels (unstructured, inverse globular, globular, and spinodal) (n = 4 images from two independent samples). b) Cell migration pattern analysis from confocal images on various microstructured hydrogels (i) non structured, (ii) inverse globular, (iii) globular, and (iv) spinodal. The black line indicates the hydrogel surface (n = 4 images from two independent samples). Scale bar indicates 5 µm. c) Representative normalized fluorescent intensity calculation at the hydrogel‐cell interface (n = 4 images from two independent samples). d) Number of cells that migrated into the hydrogels (n = 2 independent samples). e) Schematic overview depicting phase separation induced microstructurization in ATPS hydrogels, the obtained tunable, and various microarchitectures with a correlative perspective of their mechanical properties and cell migration capability due to differently structured internal micro interfaces. ^*^ indicates statistical significance at *p*<0.05, as determined by Kruskal–Wallis ANOVA. All data are presented as mean ± s.e.m.

To demonstrate as proof of concept that distinct ATPS sculpted microarchitectural configurations can distinctly influence biological function, we investigated the impact of hydrogel architecture on cell migration. While hydrogel pore size is determinant for biological activity,^[^ ^38^, ^39^ ^]^ the mechanisms by which internal pore structures emerge remain underexplored. We hypothesized that different microstructural features could benefit cellular migration by dictating pore formation. Hence, we examined MCF‐7 cell migration across various ATPS microarchitected hydrogels, including nanoporous, inverse globular, globular, and spinodal architectures. Fluorescence imaging 24 h post‐seeding revealed significantly enhanced cell migration in spinodal hydrogels compared to other configurations (**Figure** **5**a), as quantified by fluorescence intensity and migration distances (Figure 5b). Spinodal hydrogels exhibited a two‐fold increase in fluorescence intensity at the hydrogel interface compared to non‐structured and inverse globular hydrogels five days post‐seeding (Figure 5c). Quantitative cell migration analysis over a defined area demonstrated higher cell counts in spinodal hydrogels (e.g., ≈12 cells per 100 µm^2^) even when compared to other hydrogels, such as inverse globular hydrogels (Figure 5d). Migration length analysis revealed that spinodal hydrogels exhibited migration lengths of up to ≈80 µm from the hydrogel interface within five days post‐seeding (Figure S11, Supporting Information). By sculpting microarchitectural features through ATPS, and particularly for spinodal designs, we demonstrated enhanced cellular migratory potential, offering a material system that could potentially be explored to engineer living matter (Figure 5e).

## 3 Conclusion

This work demonstrates a new strategy to tailor hydrogel performance by shaping their internal microarchitecture through ATPS. We demonstrate that the improvements in stiffness, toughness, and energy dissipation arise directly from the specific microarchitectures (i.e., globular, inverse globular, and spinodal structures) as well as local polymer enrichment rather than from network interpenetration. Spinodal designs, in particular, provide exceptional mechanical advantages by distributing loads efficiently and dissipating stress in elastic hydrogels. To extend this concept, we explored single‐polymer phase separation (SPPS) as a novel approach in which controlled salt addition drives phase separation, eliminating the need for dual polymers. This method reliably generates well‐defined architectures while reducing polymer usage, offering a streamlined, scalable, and cost‐effective pathway to control mechanics. Altogether, the study emphasizes that internal micro architecture is a primary determinant of hydrogel behavior and introduces a broadly applicable framework for designing materials with tunable mechanical properties for material science‐driven diverse fields.

## 4 Experimental Section

### Materials

Alginic acid sodium salt was obtained from Sigma–Aldrich (USA), Polyethylene Glycol Dimethacrylate was sourced from TCI (Tokyo Chemical Industry, Japan), and Gelatin methacrylate was synthesized as reported previously.^[^ ^40^ ^]^ Briefly, 10% w/v of porcine Gelatin type A (Sigma–Aldrich) was dissolved in prewarmed PBS after constantly stirring at 240 rpm and 50 °C until completely dissolved. 8% v/v methacrylic anhydride (Sigma–Aldrich) was patiently added in a dropwise manner while constantly stirring for 2 h. An equal volume of preheated PBS was added to the solution and was briefly stirred until homogeneous. The solution was subsequently dialyzed in 12–14 kDa cutoff dialysis membranes (Thermo Fisher) in de‐ionized water at 40 °C for at least 5 days. The following day, an equal volume of prewarmed DI‐water was added to the mixture, and consequently, the total solution was filtered through an Express Plus filtration system (0.22 µm; Millipore) and stored at −80 °C for at least 2 days before lyophilizing. After a five‐day period of lyophilizing, freeze‐dried white foam GelMA was obtained. Phosphate‐buffered saline (PBS) without Calcium Chloride, Calcium Chloride Dihydrate, Sodium Chloride, and alginate lyase was obtained from Sigma–Aldrich (USA), alginate Rhodamine (Creative PEGworks). 99% ethanol was sourced from Boom B.V. (The Netherlands) and further diluted to 70% ethanol.

### Cloud Point Titration

The binodal curves of PEGDMA and alginate were established via cloud‐point titration following a previously reported protocol.^[^ ^25^ ^]^ Briefly, a glass vial was weighed, 2.5% alginate solution was added to the vial and weighed, while the solution was stirred, and 100% PEGDMA solution was slowly added to this mixture in a dropwise manner. After an opaque solution was obtained and maintained for more than 10 s, the glass vial was weighed once again. Hereafter, PBS was added to the solution until a transparent solution was obtained and maintained again. The glass vial was weighed, and these steps were repeated until no change in the system could be observed anymore. The amount of PEGDMA and PBS added was monitored by gravimetric analysis. The binodal curve was established by titrations, and a Merchuk fit was used to confirm that the data fitted the obtained binodal curve.

### PEGDMA‐Alginate Hydrogels

Alginate was made by mixing alginic acid sodium salt powder with PBS in a glass cuvette, overnight at room temperature (RT). Stock concentrations of 2.5%, 5% and 7.5% wt were made. Alginate 2.5% was mixed with various percentages of PEGDMA, PBS, and 0.1% wt Irgacure 2959 to attain the final concentrations. Volume scans were done by adding volume ratios x/y, with x = PEGDMA parts/total parts and y = alginate parts/total parts from various stock concentrations. For the remaining combinations of PEGDMA and alginate hydrogels, a 70/30 volume ratio was maintained unless otherwise mentioned. Silicone molds were made by using a KAI Biopsy punch 8 mm in diameter to punch through a 1 mm‐thick silicone sheet. A square was cut around the punched‐out hole for ease of use, which was placed in a CELLSTAR 12w Suspension Plate. Polymer solutions were vortexed prior to adding the required volume of the solution to the molds. The polymer solutions inside the molds were initially photo‐crosslinked by irradiating the sample at 70 mW cm^−2^ with λ = 365 nm for 80 s using a UV light source (Hamamatsu Lightning Cure LC8) to undergo free radical polymerization of the methacrylate groups of PEGDMA, followed by ionic crosslinking of alginate by immersing the sample in 100 mm of CaCl_2_ for 30 min. The obtained samples were soaked in PBS overnight prior to any measurements.

### Salt‐Induced Phase‐Separated PEGDMA Hydrogels

To identify salt‐induced phase separation of PEGDMA hydrogels, salt solutions of 20, 50, and 100 g L^−1^ NaCl were used in combination with PEGDMA polymer solution. Briefly, the photoinitiator (PI), PEGDMA prepolymer solution, and the corresponding salt solutions were added to obtain a final concentration of 0.1% (PI), 25% (PEGDMA), 20, 50, or 100 g L^−1^ of NaCl. The obtained mixture of solution was ejected into the silicon molds and photo‐crosslinked by irradiating the sample at 70 mW cm^−2^ with λ = 365 nm for 80 s using a UV light source (Hamamatsu Lightning Cure LC8).

### Holotomography Imaging

Samples were made by adding 20 µl of prepolymer solution on top of a 13 mm diameter glass cover slip in a CELLSTAR 24w Suspension Plate. Another 13 mm diameter coverslip was placed on top to form a thin hydrogel layer. The polymer solution was polymerized as mentioned earlier by covalent and ionic crosslinking of PEGDMA and alginate, respectively. The samples were then washed three times with PBS and left to soak overnight in PBS before imaging. Prior to imaging, the top coverslip of the coverslip sandwich was carefully removed, and the sample was placed onto a Tomodish (Tomocube) with the thin hydrogel layer facing the Tomodish's surface to ensure stability during imaging. A droplet of PBS was applied onto the remaining cover slip until a dome‐shaped meniscus was formed. The region of interest was then brought to focus, and a voxelated scan of 165 µm x 165 µm x 66 µm (x, y, and z, respectively) was conducted. High‐resolution 2D sections (lateral xy plane) of the optimal focal plane across the acquired data set were post‐processed in ImageJ (see section “ImageJ”). 3D reconstructions were generated using TomoAnalysis software by selecting distinct ranges of refractive indices to form a refractive index intensity map. For PEGDMA‐alginate and PEGDMA‐GelMA microarchitectured phase‐separated samples, this range started at 1.337–1.340 with the lightest yellow hue. Higher refractive index ranges were represented by progressively deeper shades of orange and red, corresponding to increasing values of refractive index in increments of 0.005. For salt‐induced phase separating samples, the range started at 1.335–1.340 with the lightest yellow hue. Non‐separated samples were made visible by including a lower refractive index range resembling the refractive index of water (1.333–1.335).

### Rheological Characterization

The rheological and mechanical properties of the hydrogels were characterized using an HR20 rheometer (TA Instruments, USA) equipped with an 8 mm Peltier parallel plate geometry. All measurements were conducted at a controlled temperature of 25 °C. Hydrogel samples for rheological testing were prepared by pipetting the required prepolymer solution into an 8 mm diameter silicone mold, yielding a 1 mm thick hydrogel disc. Samples were positioned on the rheometer, and an 8 mm Peltier plate was lowered onto the hydrogel until an axial force of 0.05 N was reached, ensuring proper contact between the hydrogel and the rheometer's plate. Storage (G′) and loss (G″) moduli were obtained through a frequency sweep at a fixed strain of 1% over a frequency range of 0.15 to 15 Hz (corresponding to an angular frequency of 1 to 100 rad/s), measuring 5 points every decade. All measurements were performed at the linear viscoelastic region of the tested hydrogels. Compression tests were conducted at a constant linear displacement rate of 10 µm^−1^s, with data acquisition at 1 s intervals. Stress–strain graphs were plotted, and the compressive modulus was calculated using the slope of the linear region in the stress–strain curve. The toughness was calculated from the area under the curve, and the fracture stress was plotted as the stress at the breaking point of the samples.

### Energy Dissipation

Samples were prepared as described previously. Samples were soaked in 5 U mL^−1^ alginate lyase overnight. Hereafter, samples were washed at least 3 times with PBS to remove any remaining alginate lyase. Energy dissipation of hydrogels was analyzed with the HR10 rheometer (TA Instruments, USA) and similar rheological equipment. Samples were positioned on the rheometer, and dehydration was prevented using hydrated wipes placed around the sample. Subsequently, a ring of silicon oil was placed around the hydrogel at a distance of 1 centimeter from the hydrogel. To assess the energy dissipation rate of the hydrogels, the 8 mm Peltier plate was lowered at a displacement rate of 100 µm^−1^s until 15% of the hydrogel's height was compressed. Normal strain was kept constant on the hydrogel during the measurement, as stress was measured for 1000 s or until the samples collapsed. Normal stress values were normalized by dividing by the highest measured value of the sample. Energy dissipation rate was derived from the negative of the slope at (0.8 to 1.2 s).

### Tensile Testing

Tensile measurements were performed using a Zwick/Roell Z0.5 TN Universal Testing Machine (Zwick/Roell, Ulm, Germany) equipped with a 100 N load cell. Hydrogel samples were prepared using a pre‐made dumbbell‐shaped silicon mold with a width of 4 mm, a thickness of 1 mm, and a gauge length of 20 mm. The tensile tests were performed at a loading speed of 5 mm min^−1^ until failure, with a preload of 0.1 N and gauge length correction applied. Tensile strength and Young's modulus were calculated based on the maximum stress at failure and the initial slope of the stress–strain curve, respectively. Each test was carried out in triplicate. Cyclic tensile tests were performed at a loading speed of 20 mm min^−1^, with incremental load steps of 0.1 N for up to 20 cycles or until sample failure. A preload of 0.01 N and gauge length correction were applied. The maximum number of cycles withstood by the sample and the maximum force at failure were recorded and plotted.

### Cell Culture & Migration Studies

MCF7 cells were cultured in DMEM supplemented medium with 10% FBS, 1% penicillin/streptomycin until they reached a confluency of 80% with medium refreshed every two days. The confluent cells were harvested and used for migration studies. Prior to cell seeding, polymer precursor solutions were injected in the middle inlet of a µ‐slide chemotaxis ibiTreat chip (Ibidi GmbH) until the middle channel of the chip was filled and subsequently polymerized by UV light to obtain respective micro‐architected (globular, inverse globular, and spinodal) and non‐architected hydrogels. Harvested MCF7 cells were injected into the chip's outer inlets and were allowed to attach for 4 h and then imaged for cell migration into the middle channel using an EVOS fluorescence microscope at various time points (0, 24, 120 h).

### Image Analysis and Statistics

Images were analyzed with FIJI ImageJ software. Strut width was measured using ImageJ. Samples were further thresholded, and particles were analyzed by ImageJ's default threshold. Feret's diameter, Sphericity, Circularity, and Roundness were calculated by ImageJ. Data was analyzed using TRIOS software, Microsoft Excel and OriginPro 2019b. Graphs, curve‐fitting, and statistical analysis were done using OriginPro 2019b. All experimental groups included at least three samples (n = 3). Statistical significance was expressed by an asterisk “^*^” for p< 0.05, or n.s. for when no significance was observed. Illustrations and schematics were designed in Blender, Adobe Illustrator, and Biorender.

## Conflict of Interest

The authors declare no conflict of interest.

## Supporting information



Supporting Information

## Data Availability

The data that support the findings of this study are available from the corresponding author upon reasonable request.
