# Supplementary material for: Arresting Microphase Separation Encodes Material Mechanics by Sculpting Microarchitectures and Local Polymer Enrichment
Source: Adv Mater. 2025 Dec 10;38(20):e12578. doi: 10.1002/adma.202512578 (PMC13054115; doi:10.1002/adma.202512578)
Supplement: Supplementary file 1 — Supporting Information [file ADMA-38-e12578-s001.docx]

**Supporting information**

**Arresting microphase separation encodes material mechanics by sculpting microarchitectures and local polymer enrichment**

*Castro Johnbosco ^a#^, Floris Dalenoord ^a#^, Jarno Hiemstra ^a^, Yu Na ^a^,* *Cecile Bosmans^a^, Christine Gering^a^, Niels Willemen ^a^, Su Ryon Shin ^b^, and Jeroen Leijten ^a*^*

^a^ Leijten Laboratory, Department of BioEngineering Technologies, TechMed Centre, Faculty of Science and Technology, University of Twente, The Netherlands.

^b^ Division of Engineering in Medicine, Department of Medicine, Brigham and Women's Hospital, Harvard Medical School, Boston, MA 02139, USA.

*^#^ Authors contributed equally.,*

*^*^corresponding author: [jeroen.leijten@utwente.nl](mailto:jeroen.leijten@utwente.nl)*

*Keywords: Aqueous two-phase system, multiscale materials, soft matter, mechanics, microarchitectures*


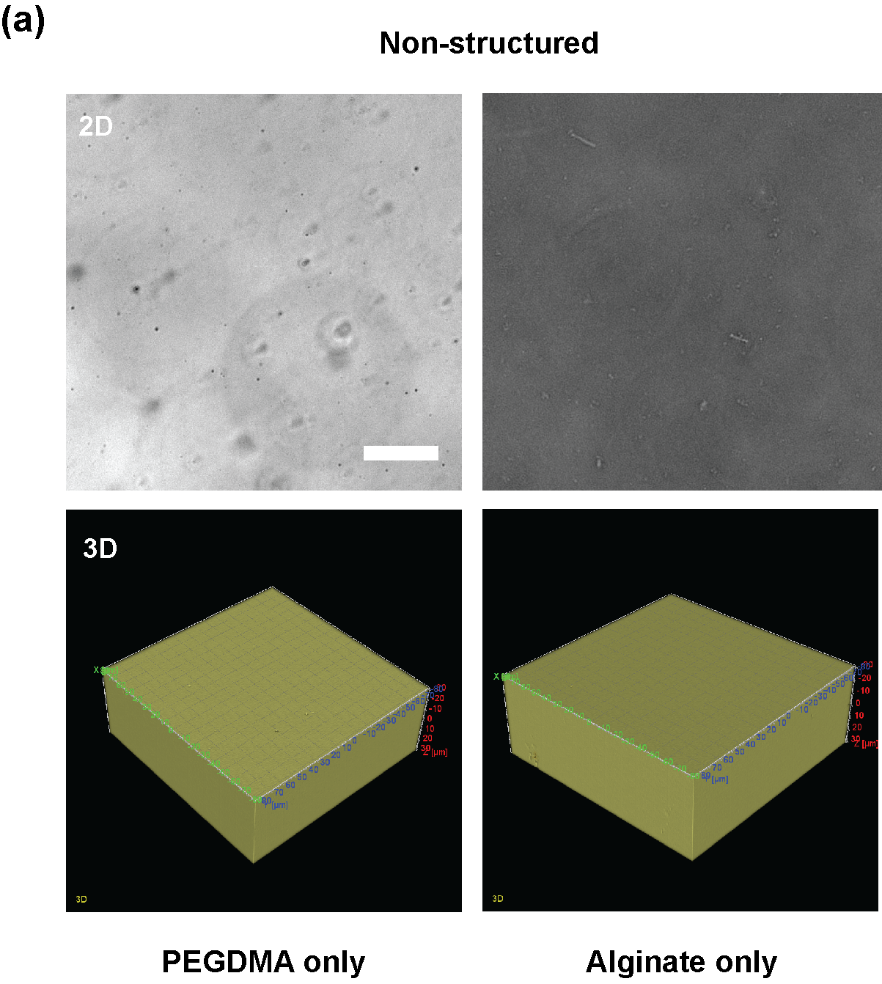


*Figure S1: Representative holotomographic images PEGDMA only and alginate only in 2D and 3D denoting the absence of microarchitectures when unmixed non-phase separated. Scale bar indicates 50µm*

*
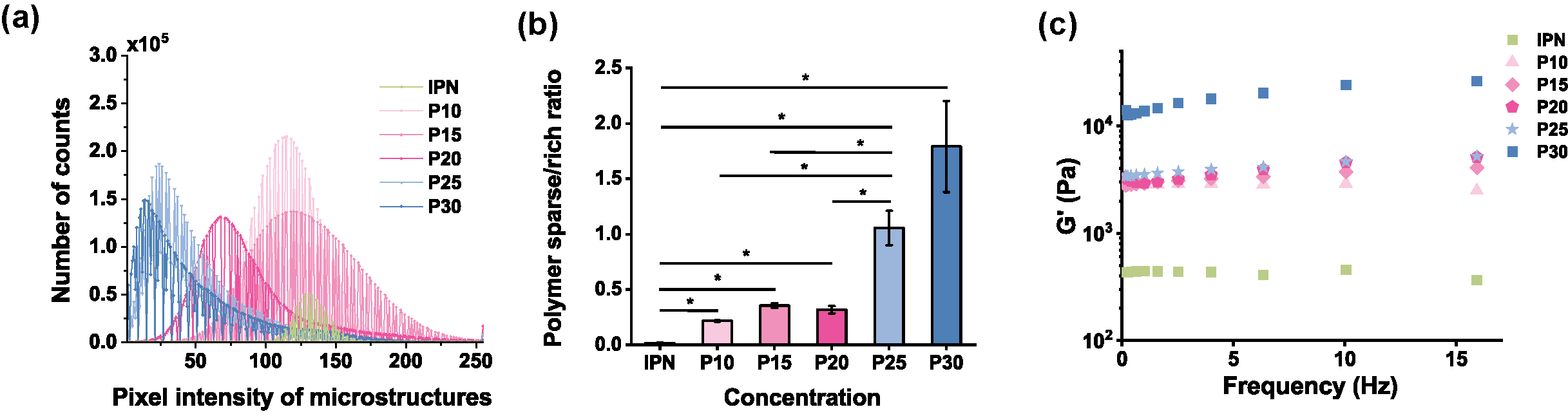
*

*Figure S2: (a) Intensity mapping and the representative number of intensity point counts calculated per pixel/image (n=3). (b) Intensity ratio of polymer sparse/rich regions which represents the binodal distribution of alternating polymer sparse and rich regions. The lower value denotes polymer miscibility, and the higher value represents polymer immiscibility with distinct phase separated regions (n=3). (c) Frequency sweep on various microstructured hydrogels to determine storage modulus (G') (n=3)*

*
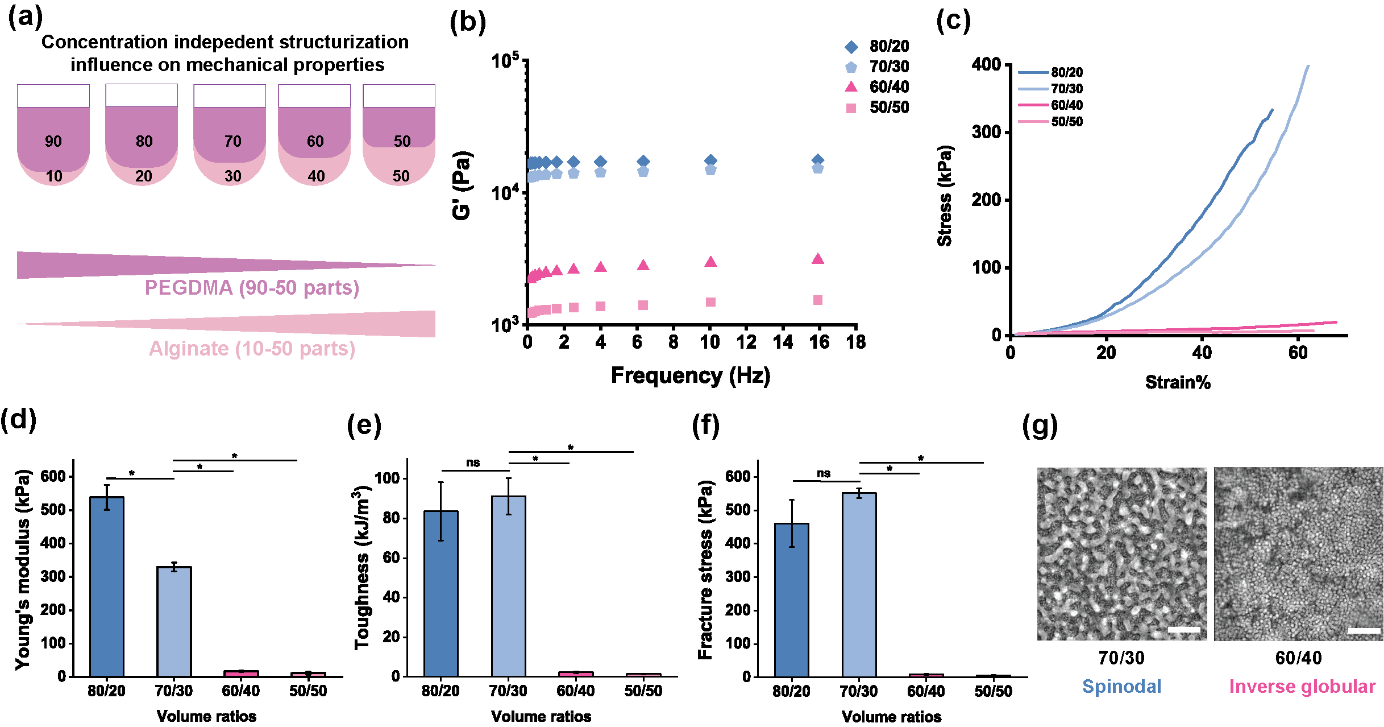
*

*Figure S3: Characterization of polymer blends' concentration independent micro structurization and their influence on mechanical properties, a) Schematic depiction of polymer immiscibility based on different volume ratios governing phase separation without changing the concentration of PEGDMA and Alginate. (b) Frequency sweep on various hydrogels produced by varying volume ratios(PEGDMA/Alginate of 80/20 to 50/50) to determine the representative storage modulus (G'). (c) Representative stress-strain curves of hydrogels with varying volume ratios are used to obtain the yield stress of microarchitected hydrogels. (d) Young's modulus, (e) Toughness, and (f) Fracture Stress of hydrogels produced using various polymer ratios of (PEGDMA/Alginate) from 80/20 to 50/50 (n=3). (g) Holotomographic micrographs of 70/30 ratio yielding spinodal and 60/40 ratio yielding inverse globular microarchitectures. (n=3 samples per condition).* indicates statistical significance at p<0.05, as determined by Kruskal–Wallis ANOVA. All data are presented as mean ± s.e.m.*

*
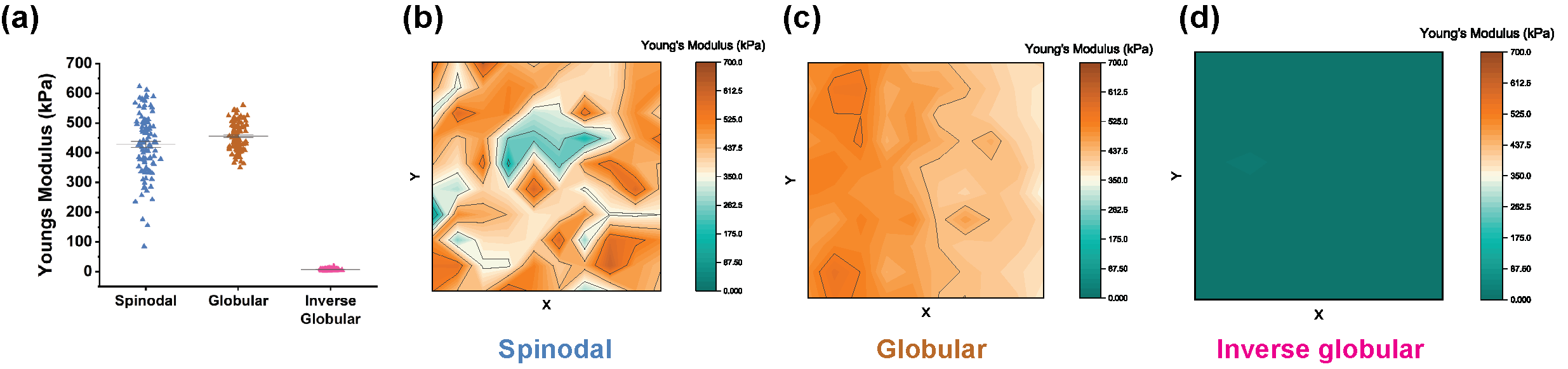
*

*Figure S4: Nano indentation analysis on hydrogels sections with differently sculpted microarchitectures. (a) Young’s modulus of spinodal, globular and inverse globular hydrogels (n=100 data points from 3 independent samples). Representative topographical mechanical mapping of 10x10 matrix scan to determine the Young’s modulus on sculpted (b) spinodal, (c) globular and (d) inverse globular hydrogel sections. (n=3 samples per condition)*

*
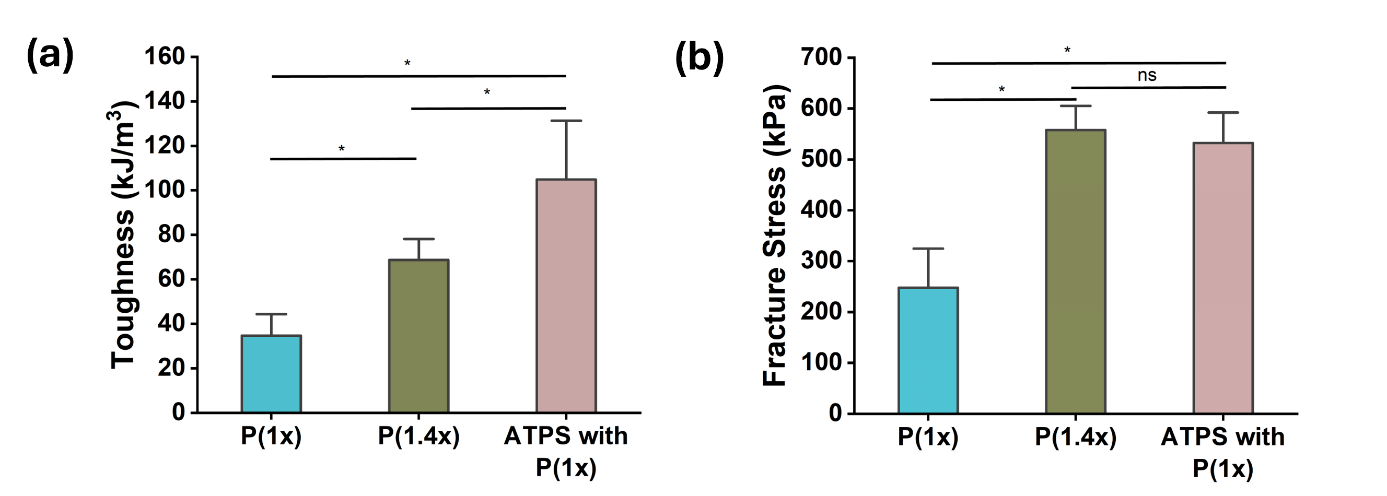
*

*Figure S5: Rheological measurements of the (a) toughness, (b) fracture stress of spinodal PEGDMA hydrogels as well as nanoporous PEGDMA hydrogels of which the polymer concentration was either matched to that of the local polymer enrichment (1.4x; 25%) or total polymer content (1x; 17.5%) of the spinodal PEGDMA hydrogel*

*
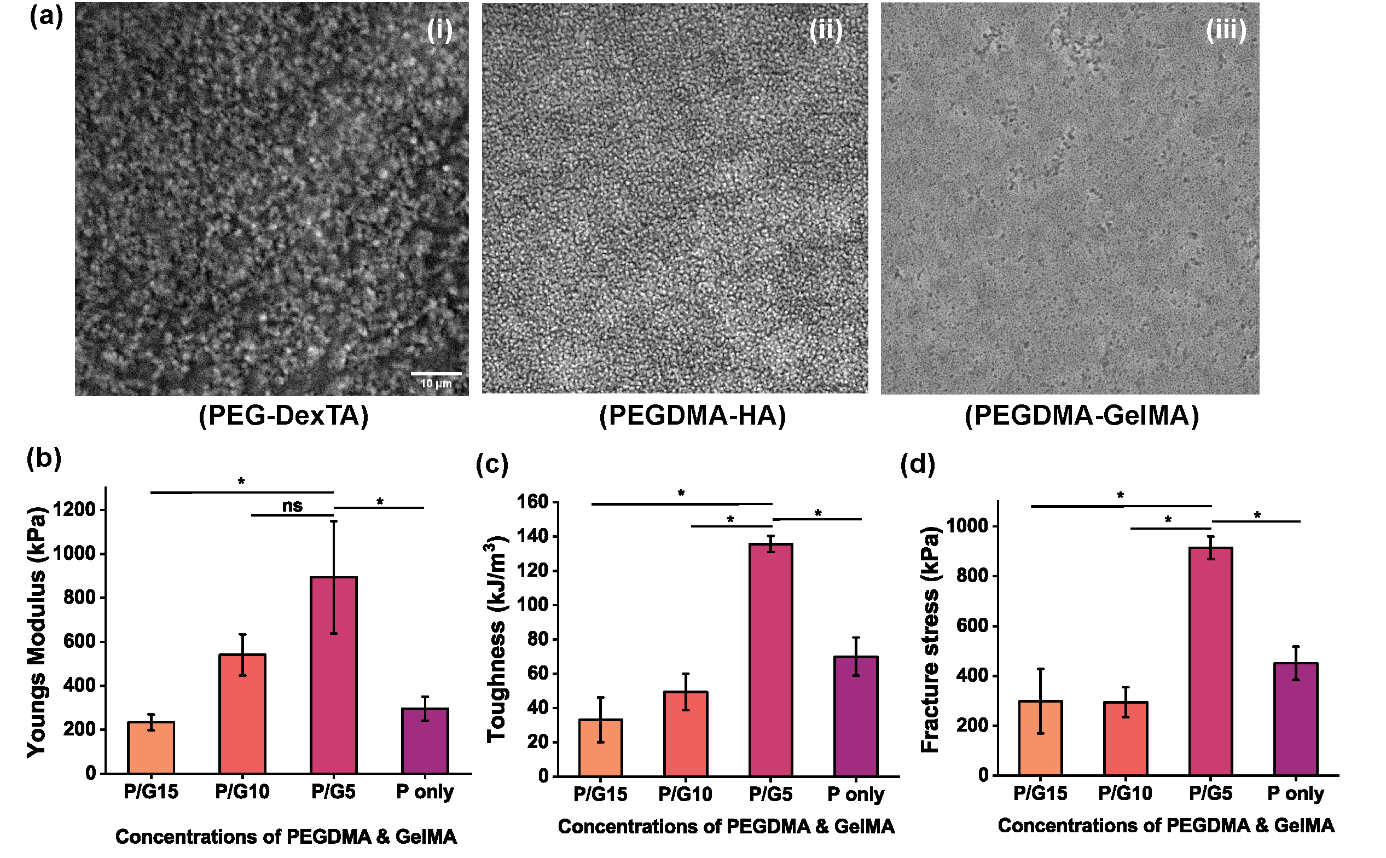
*

*Figure S6: (a) Holotomographic images of micro architected hydrogels using various biopolymer formulations that facilitate phase separation. (a) (i) PEG and Dextran conjugated with tyramine (DexTA), (ii)PEGDMA and hyaluronic acid, (iii) PEGDMA and GelMA. To investigate whether the effects of microarchitecture formations are material dependent, gelatin methacrylate (GelMA) was used in combination with PEGDMA to create ATPS microarchitected hydrogels using GelMA 5% with PEGDMA 25% in a volume ratio of 70/30. Rheological measurements of (c) Young’s Modulus, (d) toughness, and (e) fracture stress of GelMA- PEGDMA phase separated microarchitecture. (n=3 samples per condition). ).* indicates statistical significance at p<0.05, as determined by Kruskal–Wallis ANOVA, “ns” indicates non – significance. All data are presented as mean ± s.e.m.*

*
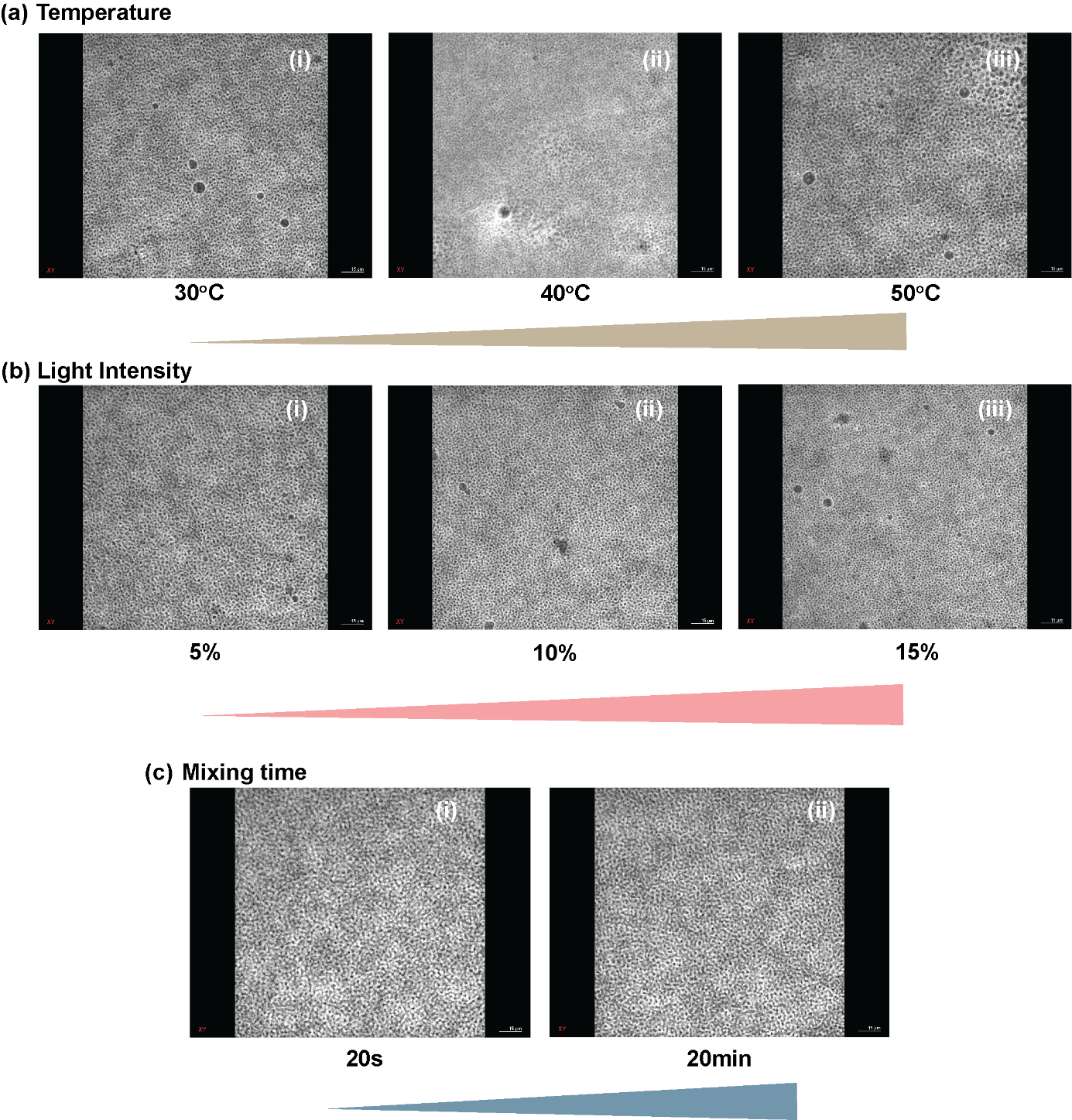
*

*Figure S7: Representative holotomographic images of micro architected hydrogels using PEGDMA 25% and Alginate 0.8% in a 70/30 ratio to determine environmental factors including temperature, light intensity and polymer mixing time. (a) Hydrogel formation at different temperatures (i) 30^o^C, (ii) 40^o^C, (iii) 50^o^C; (b) with different light intensities (i) 5%, (ii) 10%, (iii) 15%; (c) time before crosslinking (i) 20 seconds (20s), (ii) 20 minutes (20min). (n=3) samples per condition.*

*
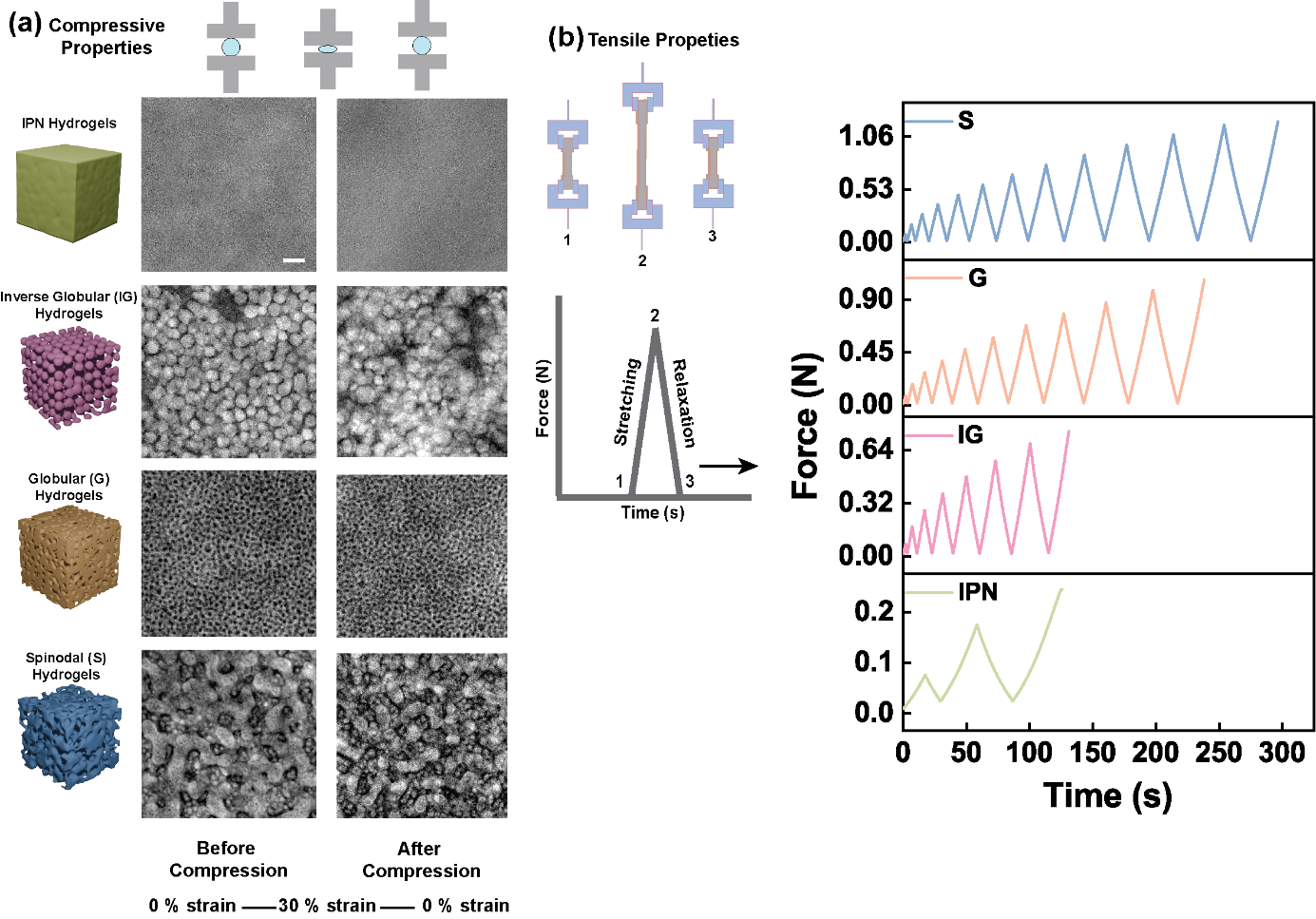
*

*Figure S8: (a) Representative Holotomographic images of various microstructure patterns of inverse globular, spinodal & globular hydrogels obtained before compression, and post 30% compression to determine the geometric recovery ability of these microstructures inside these hydrogels (n=3 individual images from 2 independent samples). (b) Cyclic stretching measurement to determine the effect of microarchitectures of hydrogels' mechanical integrity under cyclic deformation.*

*
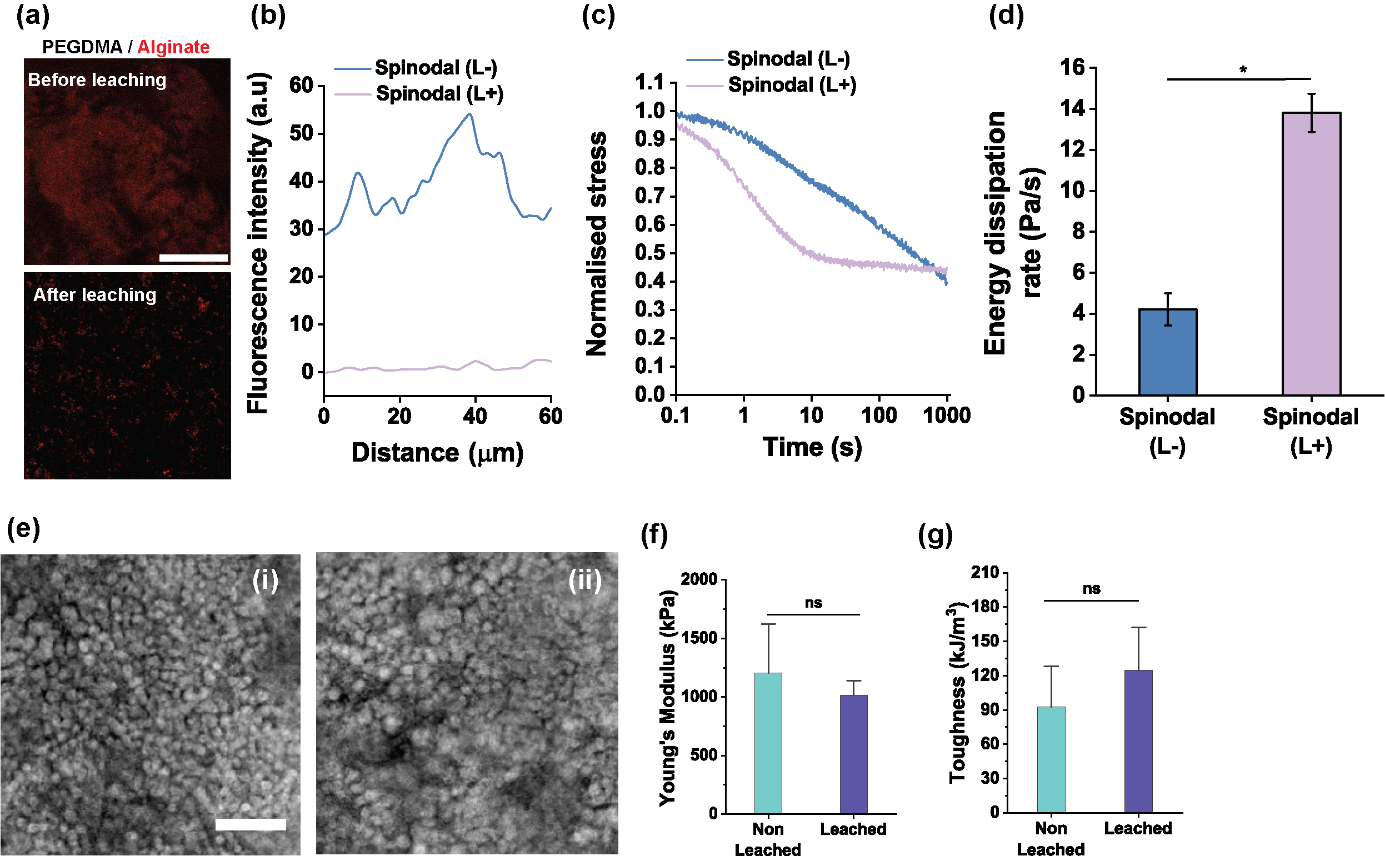
*

*Figure S9: (a) Representative fluorescent images (Scale bar : 100µm) and (b) fluorescent intensity of alginate leached and non-leached spinodal hydrogels. (c) Representative energy dissipation curves and (d) Energy dissipation rates of alginate leached and non-leached spinodal hydrogels. (e) Representative Holotomographic images of PEGDMA – Alginate inverse globular hydrogels (i) Non-leached (ii) leached alginate samples (Scale bar : 20µm). Mechanical analysis (f) Young’s modulus, (g) Toughness of alginate leached and non-leached samples.(n=3 samples per condition).* indicates statistical significance at p<0.05, as determined by Kruskal–Wallis ANOVA. All data are presented as mean ± s.e.m.*


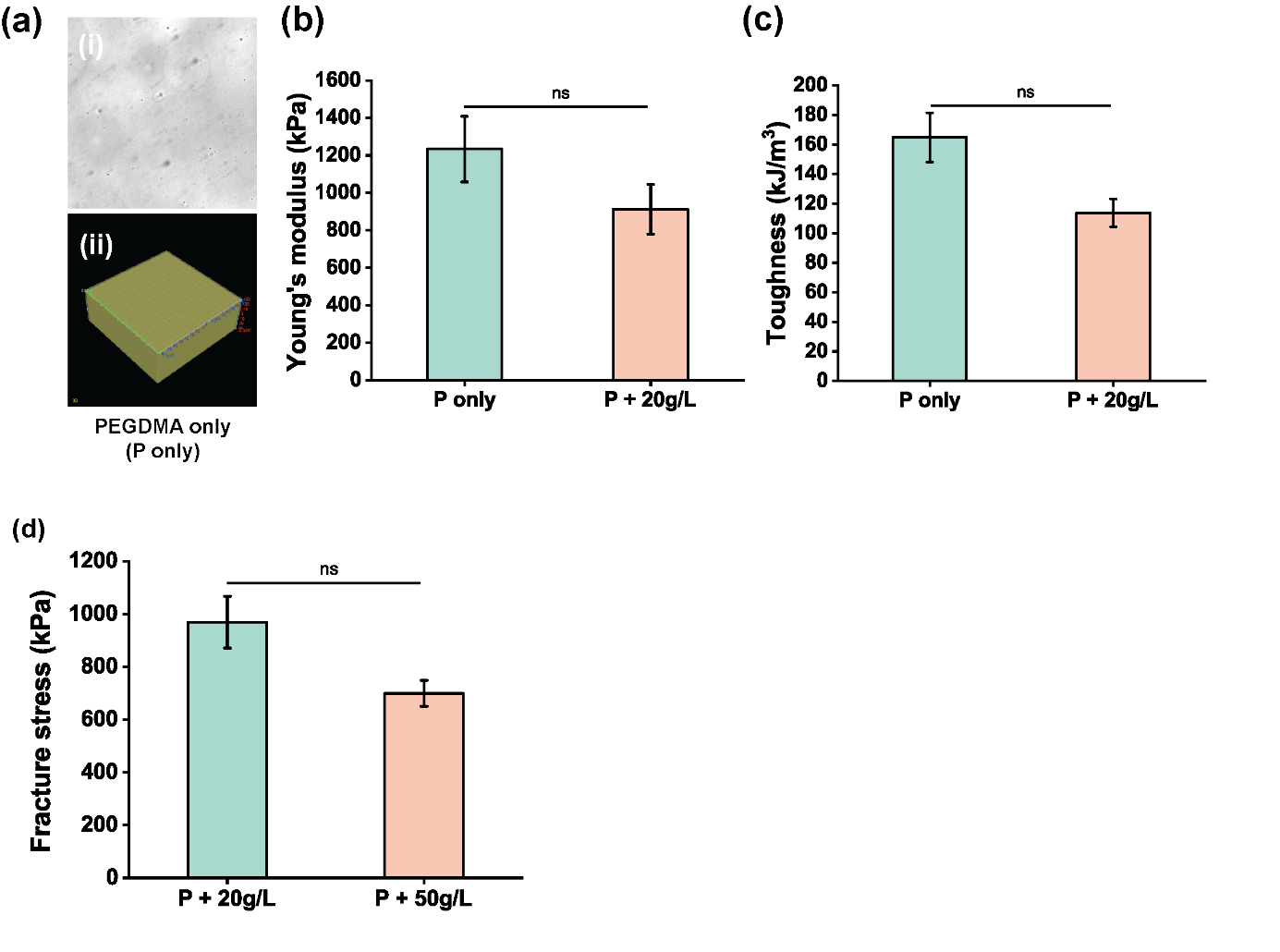


*Figure S10: (a) Holotomographic micrographs of (i) 2D and (ii) 3D of non-architectured PEGDMA only hydrogels. Mechanical properties of PEGDMA hydrogels without salt addition (P only) or PEGDMA hydrogels with 20 g/L salt added (P+20g/L) for (b) Young’s modulus, (c) toughness, (d) fracture stress. (n=3 samples per condition).* indicates statistical significance, and “ns” indicates non - significant. at p<0.05, as determined by Kruskal–Wallis ANOVA. All data are presented as mean ± s.e.m.*


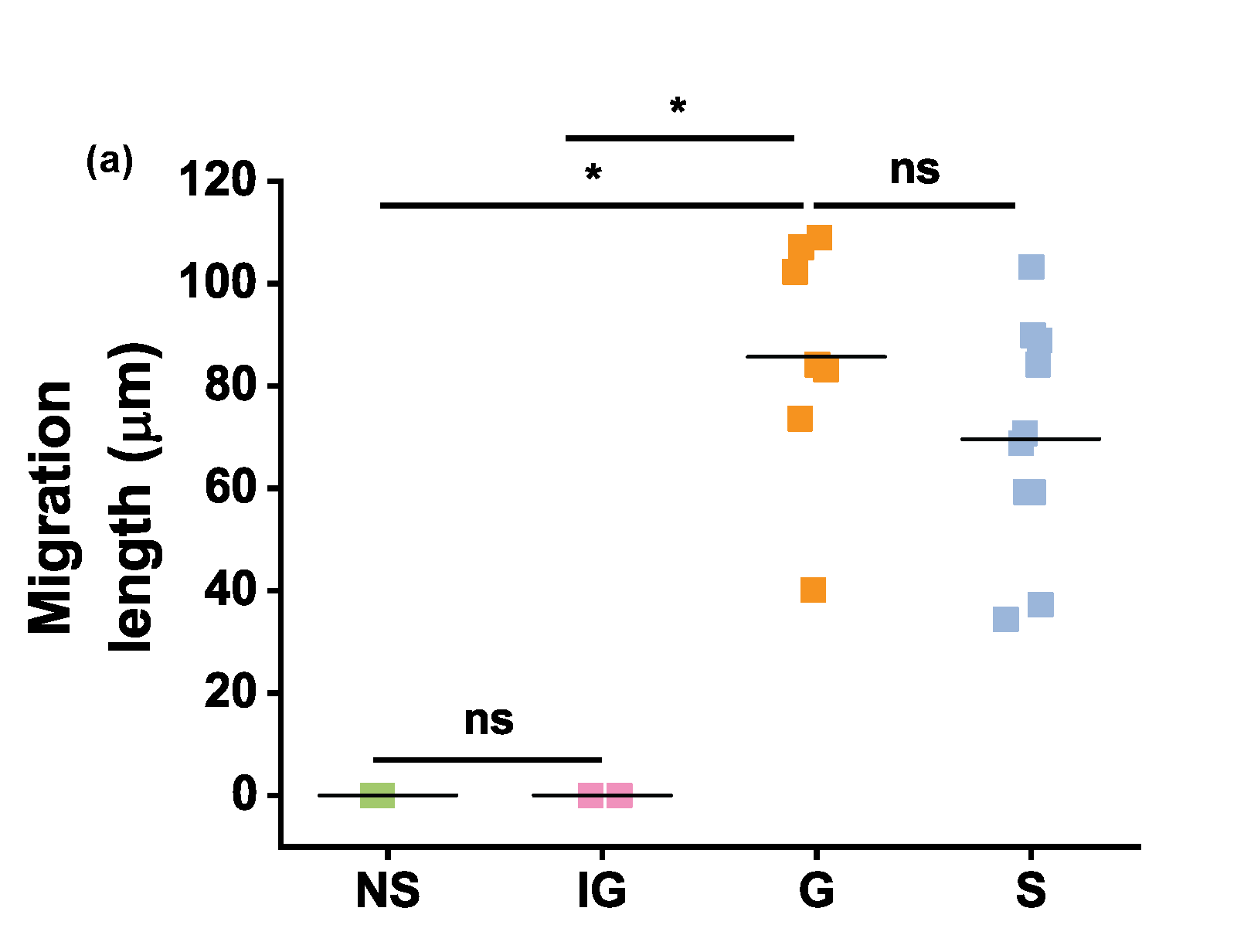


*Figure S11: Migration length of MCF-7 cells from the hydrogel-cell interface on various microarchitected hydrogels. n≥7 cells from two independent samples.).* indicates statistical significance, and “ns” indicates non - significant. at p<0.05, as determined by Kruskal–Wallis ANOVA. All data are presented as mean ± s.e.m.*


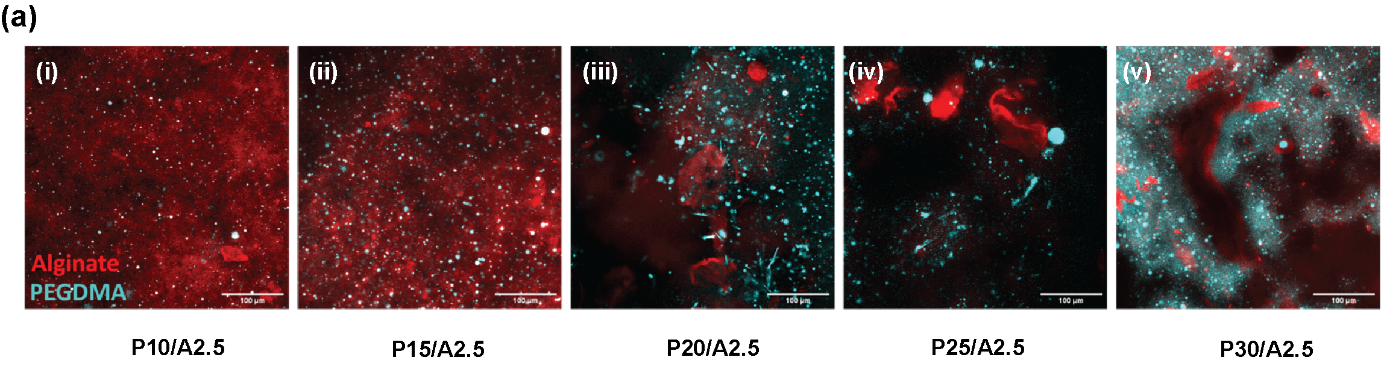


*Figure S12: Fluorescent confocal micrographs of different concentrations of PEGDMA (cyan) and Alginate (red) to determine the phase separation induced microarchitecture formation. Confocal micrographs represent the inverse globular to spinodal transformation with an increase in PEGDMA (P) concentration relative to alginate (A) concentration. Scale bar indicates 100µm.*


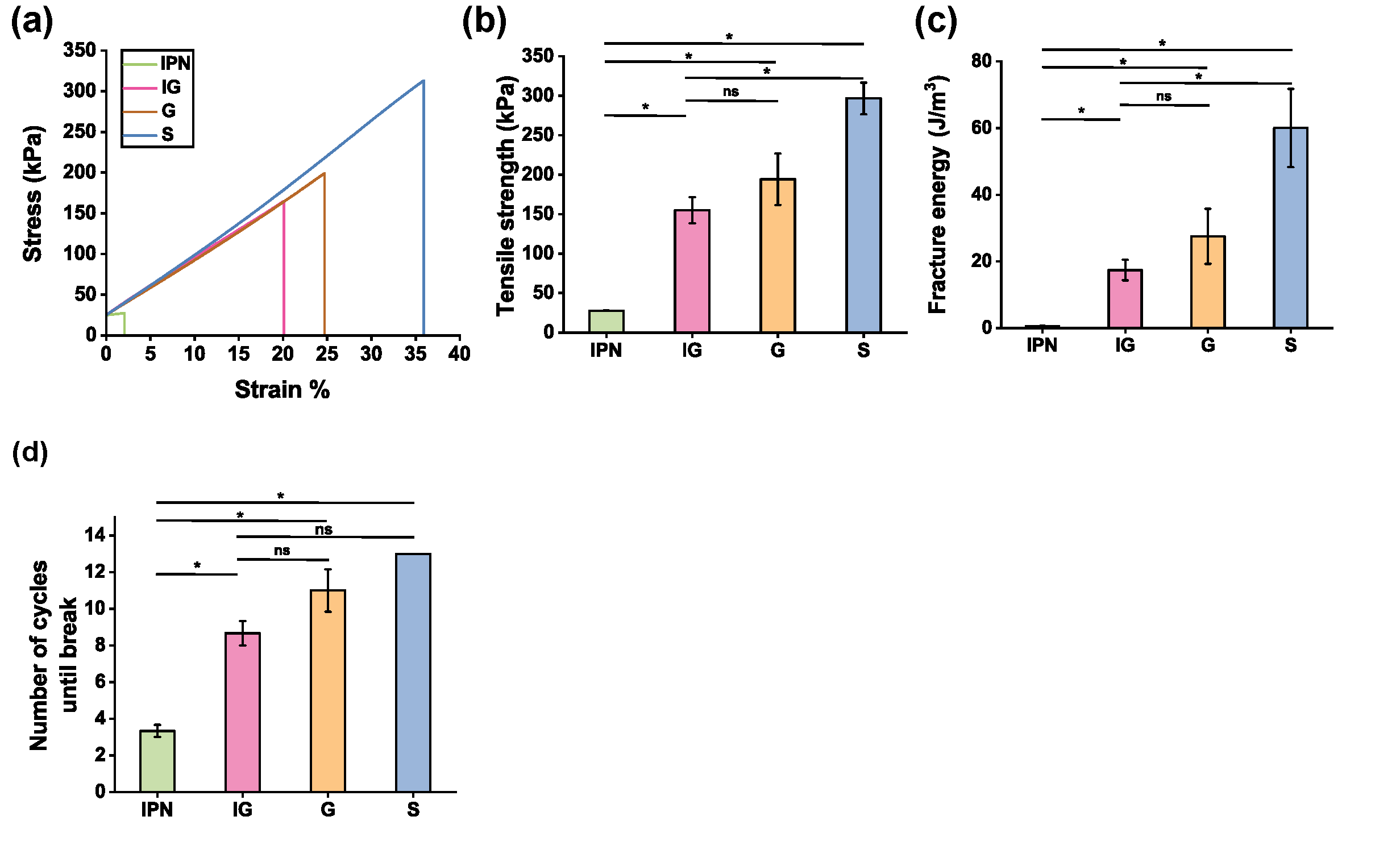


*Figure S13: Tensile measurements on microarchitectured and non-architectured hydrogels to determine (a) stress-strain relationship, (b) tensile strength, (c) fracture energy, and (d) number of cycles until breakage. n=3 samples per condition. ).* indicates statistical significance, and “ns” indicates non - significant. at p<0.05, as determined by Kruskal–Wallis ANOVA. All data are presented as mean ± s.e.m.*


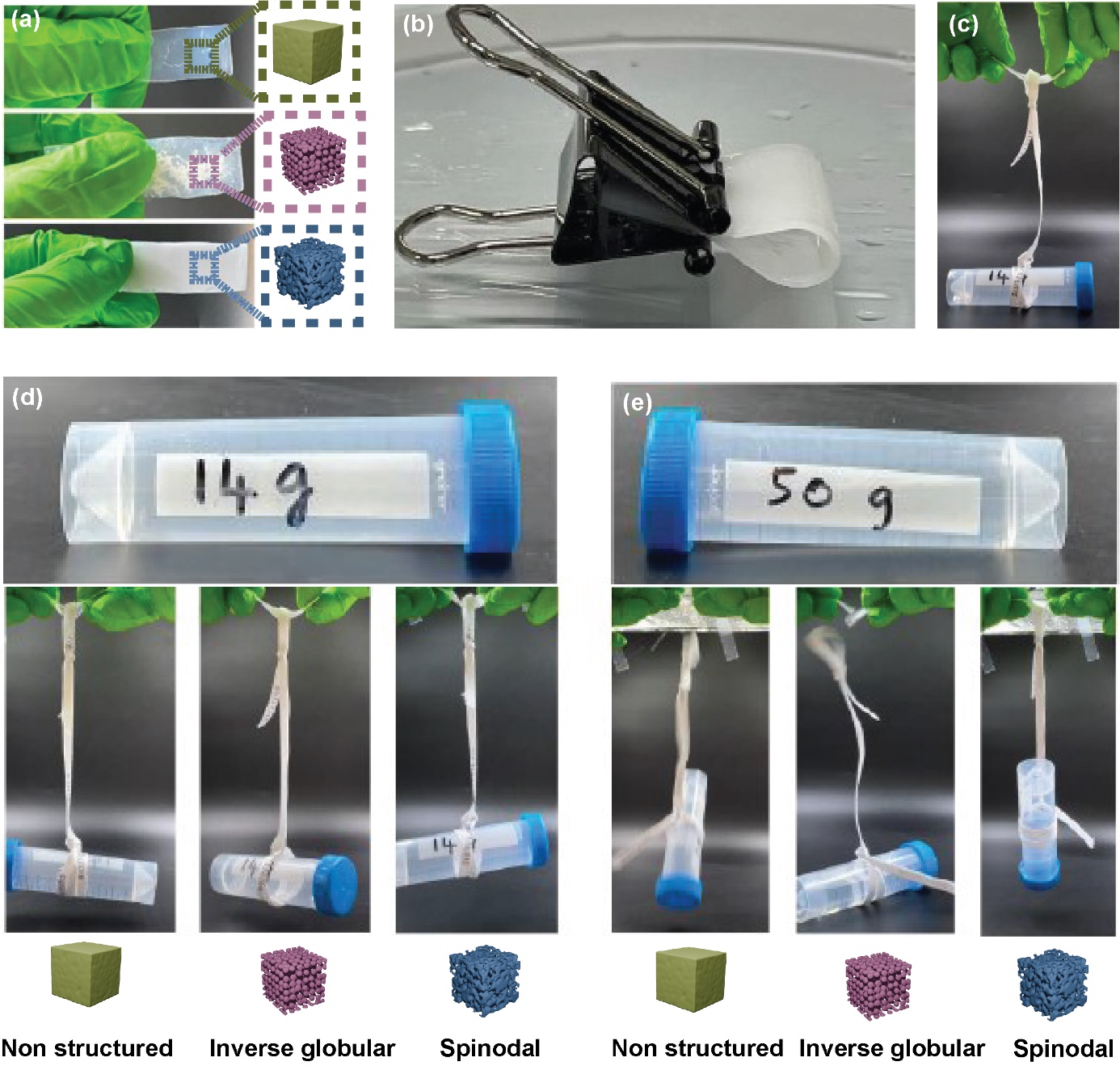


*Figure S14: (a) Macroscopic images of microarchitected hydrogels (e.g., inverse globular and spinodal) and non-structured hydrogels. (b) Bending tests were performed to reveal the flexibility of the hydrogels (spinodal hydrogels shown here); crosslinked sheets of hydrogel ends were clamped to analyze bendability and flexibility of hydrogels. (c) Weight-bearing analyses of crosslinked hydrogel sheets to determine the robustness of the microarchitectures in hydrogels. Different weights (d) 14 grams and (e) 50 grams were lifted using various hydrogel sheets (non-structured, inverse globular, and spinodal), which revealed spinodal hydrogels were sufficiently strong and tough to lift 50 grams while non-structured and inverse globular hydrogels could not withstand this weight > 14 grams. n≥3 samples per condition.*

*
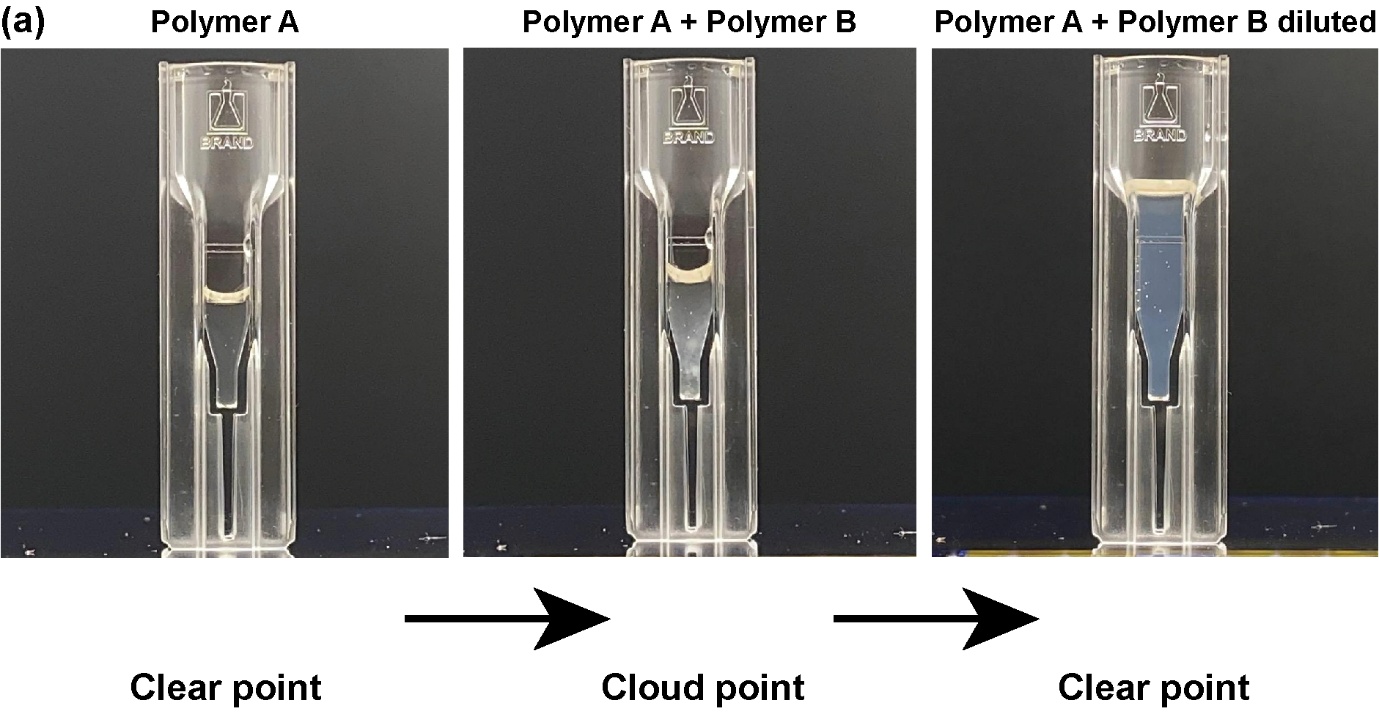
*

*Figure S15: (a) Photographs of transparent non-phase separated blend, phase separated blend, and diluted phase separated blend during cloud and clear point indicating the phase separation of two polymers at certain concentrations.*

*
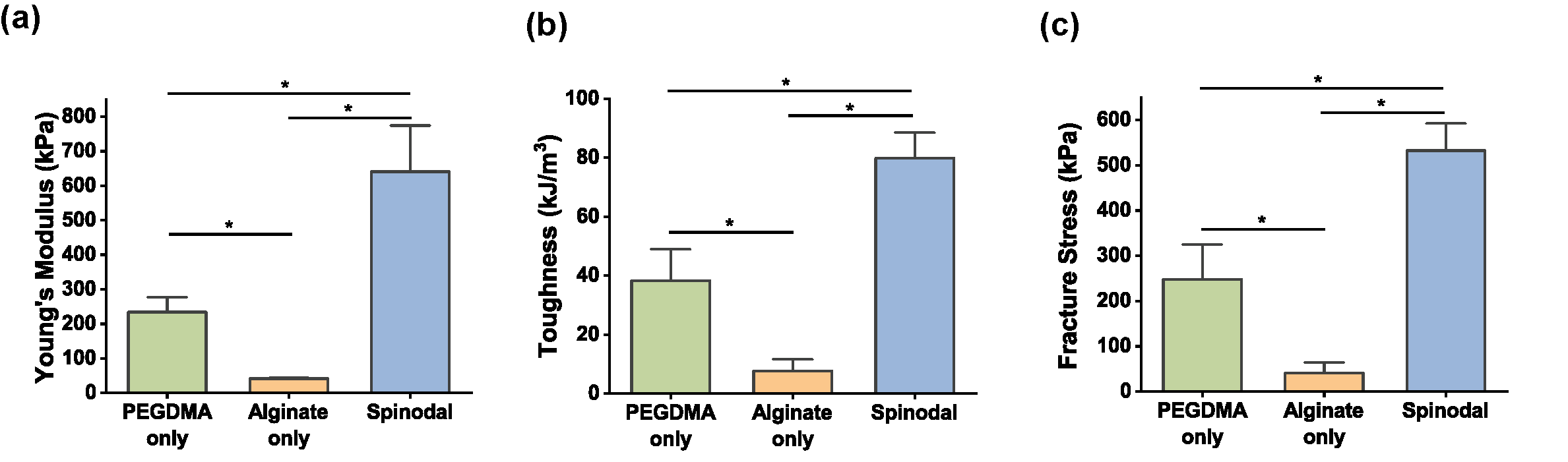
*

*Figure S16: Mechanical properties of PEGDMA only (17.5%), alginate only (0.8%) as control conditions compared to spinodal hydrogels (PEGDMA 17.5% and alginate 0.8%) to determine the (a) Young’s modulus, (b) Toughness, (c) Fracture stress. (n≥2 samples per condition). * indicates statistical significance at p < 0.05, as determined by Kruskal–Wallis ANOVA.*

*
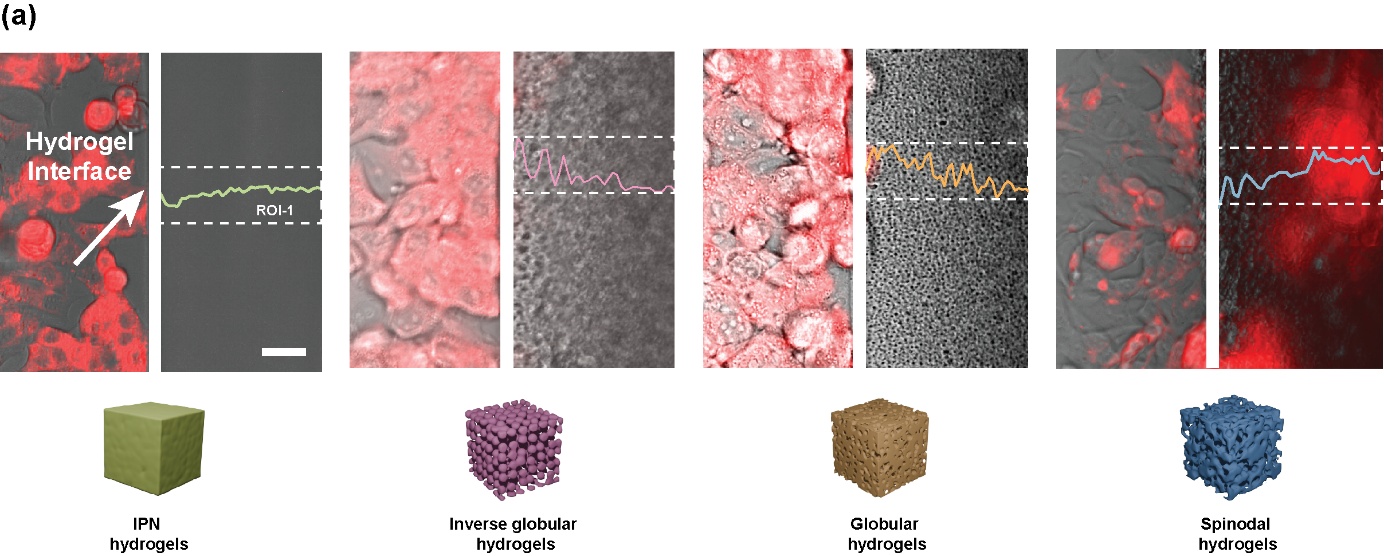
*

*Figure S17: Representative normalized fluorescent intensity at the hydrogel-cell interface overlayed on fluorescent images obtained from differently sculpted hydrogel sections indicating the analysis region. Zoomed in ROIs represents arbitrary value of intensity over the distance between the hydrogel interface and the migrated cells.*

*Methods*

*Stock Concentrations*

Alginate was prepared by mixing 50 mg, 100 mg and 150 mg with 2 ml of PBS in a glass vial stirred by a magnet fish at 300rpm at RT overnight to obtain 2.5%, 5.0% and 7.5% alginate stocks respectively. PI stock solution was made by dissolving 6 mg (Irgacure 2959) with 1000 ml PBS to obtain 0.6% PI stock solution. PEGDMA stock concentrations were prepared by diluting 100 % PEGDMA with PBS accordingly. For example, a 25% PEGDMA stock solution is made by dissolving 250 μl PEGDMA with 750 μl PBS. For samples that were covalently crosslinked, PI was substituted for PBS to obtain 0.1% in the prepolymer solution. Volume ratio samples were prepared by mixing PEGDMA/Alginate listed stock concentrations respectively.

*PEGDMA-GelMA hydrogels*

Similarly, 200 mg of freeze-dried GelMA was dissolved in 1000 μl DI water. Hereafter GelMA solution was mixed for at least at least 1 hour or until fully dissolved at 50°C while agitating at 500 rpm. A stock solution of 0.6%wt Irgacure 2959 was prepared. A volume ratio of 50:50 PEGDMA:GelMA was maintained while stock concentration of PEGDMA was varied. Samples were briefly raised until 50°C and hereafter thoroughly vortexed, whereafter directly crosslinked. Samples were left in PBS to soak overnight, before analysis. Photocrosslinking was conducted by using the photoinitiator Irgacure 2959 for PEGDMA and GelMA containing hydrogels. Samples were immediately crosslinked after vortexing polymer blends. Solutions were crosslinked in the molds as previously described.
